# Tissue-Specific Splicing of Disordered Segments that Embed Binding Motifs Rewires Protein Interaction Networks

**DOI:** 10.1016/j.molcel.2012.05.039

**Published:** 2012-06-29

**Authors:** Marija Buljan, Guilhem Chalancon, Sebastian Eustermann, Gunter P. Wagner, Monika Fuxreiter, Alex Bateman, M. Madan Babu

**Affiliations:** 1 MRC Laboratory of Molecular Biology, Hills Road, Cambridge CB2 0QH, UK; 2 Wellcome Trust Sanger Institute, Wellcome Trust Genome Campus, Hinxton, Cambridge CB10 1SA, UK; 3 Yale Systems Biology Institute, and Department of Ecology and Evolutionary Biology, Yale University, West Haven, CT 06516, USA; 4 Department of Biochemistry and Molecular Biology, Medical and Health Science Center, University of Debrecen, Nagyerdei krt 98, POB 6, H-4032 Debrecen, Hungary

## Abstract

Alternative inclusion of exons increases the functional diversity of proteins. Among alternatively spliced exons, tissue-specific exons play a critical role in maintaining tissue identity. This raises the question of how tissue-specific protein-coding exons influence protein function. Here we investigate the structural, functional, interaction, and evolutionary properties of constitutive, tissue-specific, and other alternative exons in human. We find that tissue-specific protein segments often contain disordered regions, are enriched in posttranslational modification sites, and frequently embed conserved binding motifs. Furthermore, genes containing tissue-specific exons tend to occupy central positions in interaction networks and display distinct interaction partners in the respective tissues, and are enriched in signaling, development, and disease genes. Based on these findings, we propose that tissue-specific inclusion of disordered segments that contain binding motifs rewires interaction networks and signaling pathways. In this way, tissue-specific splicing may contribute to functional versatility of proteins and increases the diversity of interaction networks across tissues.

## Graphical abstract

**Graphical Abstract:**

**Figure:**
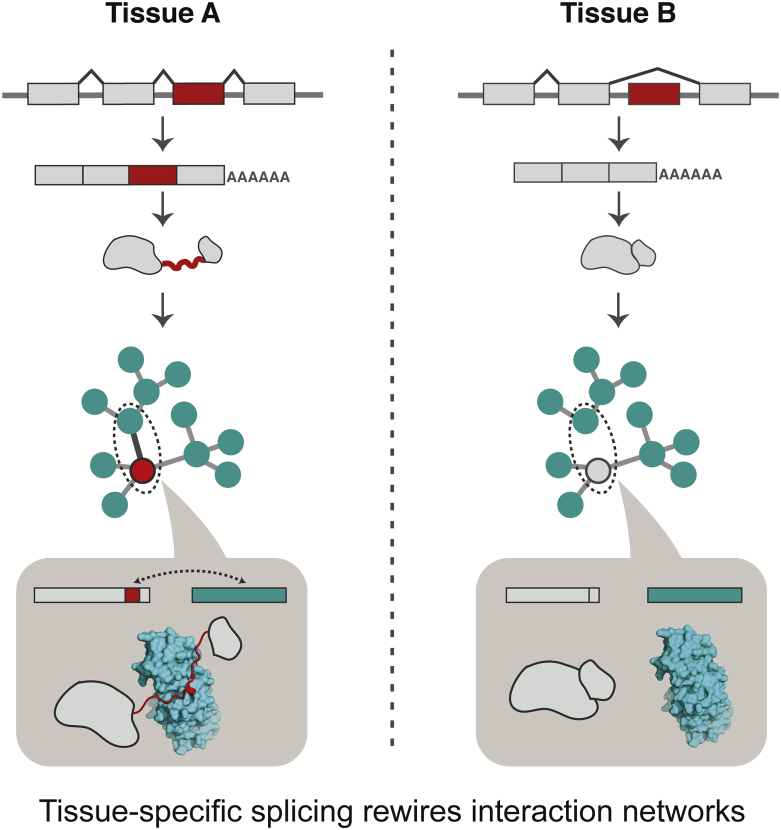

**Highlights:**

► Protein segments of tissue-specific (TS) exons frequently contain disordered regions ► TS segments contain modification sites and evolutionarily conserved binding motifs ► Genes with TS exons are hubs in interaction networks and enriched in signaling genes ► TS splicing can rewire protein networks and signaling pathways in different tissues

## Introduction

Together with gene duplication and recombination, alternative splicing plays a major role in increasing proteome diversity and organismal complexity (Keren et al., 2010; Nilsen and Graveley, 2010; Romero et al., 2006; Wang et al., 2008). In fact, current estimates suggest that nearly 90% of human genes are alternatively spliced (Wang et al., 2008). While some of the transcript isoforms are likely to be degraded by nonsense-mediated decay (Lewis et al., 2003), splicing often leads to the generation of alternative protein isoforms of the same gene (Stamm et al., 2005; Tran et al., 2011; Tress et al., 2008). Thus, on the protein level, entire segments can be inserted or deleted through the alternative splicing of protein-coding exons (Hegyi et al., 2011; Pentony and Jones, 2010; Romero et al., 2006; Yura et al., 2006). As a result, it is expected that distinct protein isoforms will often exhibit different functional characteristics (Modrek and Lee, 2002; Nilsen and Graveley, 2010; Resch et al., 2004; Stamm et al., 2005). Indeed, previous studies have offered insights into the importance of alternative splicing by investigating functional differences between isoforms from different organisms (Modrek and Lee, 2002; Nilsen and Graveley, 2010; Resch et al., 2004).

Alternative exons for which inclusion levels differ across tissues are referred to as tissue-specific (TS) exons. Studies using microarray data of mouse tissues have shown that, unlike most alternatively spliced exons, the lengths of TS exons are more often in multiples of three nucleotides (Xing and Lee, 2005b). Since differential inclusion of these exons is less likely to interrupt the frame of translation, this suggests that such exons will often have an impact at the protein level. Moreover, it is known that many TS exons play crucial roles in attaining cell identity (Kalsotra and Cooper, 2011; Witten and Ule, 2011). For instance, tissue-specific splicing plays an important role in brain and heart development (Kalsotra and Cooper, 2011), and it has been recently shown that cell-type-specific isoforms of key regulatory proteins can drive cellular differentiation (Gabut et al., 2011; Ungewitter and Scrable, 2010).

**Figure 1:**
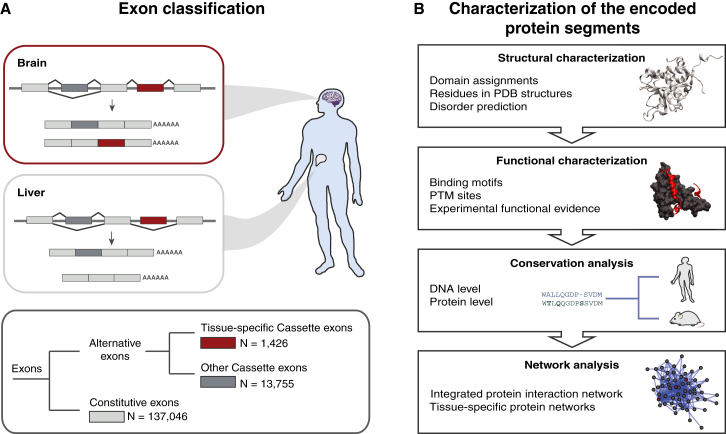
Classification of Exons and Characterization of the Protein Segments (A) Cassette exons that are tissue-specifically spliced are in red, and alternatively spliced cassette exons not present in the set of tissue-specific exons are in dark gray. Constitutive exons are in light gray. (B) Shown are types of data and computational approaches used to characterize the exons. See also Figure S1.

While individual studies have highlighted the importance of TS exons, genome-scale molecular principles by which such exons influence protein function remain to be elucidated. Toward this goal, we performed a large-scale computational analysis of constitutive, tissue-specific, and other alternative exons and their corresponding protein segments in humans (Figure 1). Our integrated analysis revealed that TS exons tend to encode protein segments that lack a well-defined structure more often than other alternative and constitutively spliced exons. Protein segments that do not adopt a well-defined three-dimensional structure are generally termed as intrinsically disordered, or unstructured regions (Dyson and Wright, 2005). In contrast, constitutive exons tend to more often map to protein domains. An analysis of the publicly available data revealed that tissue-specific protein segments are enriched to contain posttranslational modification (PTM) sites and evolutionarily conserved binding motifs. Finally, through the investigation of tissue-specific expression and protein interaction data, we observed that genes with TS exons tend to (1) occupy central positions (hubs) in interaction networks and (2) have interaction partners that are distinct in these tissues. Thus, we propose that by alternative inclusion of disordered segments, which contain binding motifs, tissue-specific splicing can rewire molecular interaction networks. Based on the observed characteristics of such segments, we delineate genome-scale molecular principles by which these segments can influence protein function and their interaction networks.

## Results

To compose a set of TS exons, we used a list of the reported cassette exons whose inclusion levels significantly differed between at least two tissue types or cell lines (Wang et al., 2008). By definition, cassette exons are either entirely included or excluded from the mature transcripts (see Figure S1A available online). We mapped Cassette exons to the known and predicted protein-coding exons in the Ensembl Database. This resulted in 1,426 protein-coding “TS Cassette exons,” for which inclusion levels were assessed in ten human tissues and five cell lines. We compared TS Cassette exons to protein-coding constitutive exons (137,046 “Constitutive exons”) and to all other protein-coding cassette exons, i.e., exons found to be alternatively spliced in mature transcripts from the Ensembl Database, but nonoverlapping with the set of TS Cassette exons (13,755 “Other Cassette exons”); see Figure 1 and the Experimental Procedures.

### Very Small Number of Tissue-Specific Exons Code for Complete Protein Domains

**Figure 2:**
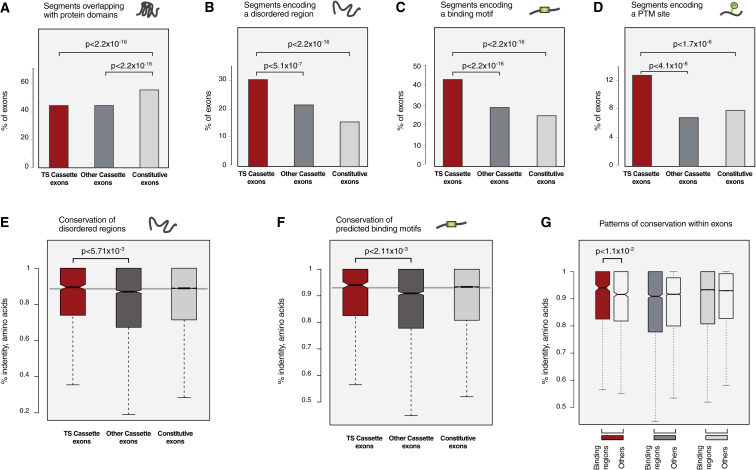
Tissue-Specific Segments Are Enriched in Disordered Binding Motifs and Are Conserved Percentages of protein segments encoded by the three different sets of exons that contain (A) Pfam domains, (B) disordered regions, (C) ANCHOR-binding motifs, and (D) PTM sites. Significance was assessed by chi-square test, and p values are indicated in each panel. Box plot of the distribution of conservation (percent amino acid identity) for the (E) disordered region, (F) binding motifs, and (G) binding motifs versus other regions for the three different sets of exons. Statistical significance was assessed using Mann-Whitney test. The median value within each set is shown with a thick black line. Boxes enclose values between the first and third quartile. Interquartile range (IQR) is calculated by subtracting the first quartile from the third quartile. All values that lie more than 1.5× IQR lower than the first quartile or 1.5× higher than the third quartile are considered to be outliers and were removed from the graphs to improve visualization. The smallest and highest values that are not outliers are connected with the dashed line. The notches correspond to ∼95% confidence interval for the median. See also Figure S2 and Table S1.

Assignment of Pfam domains to sequences showed that protein domains less frequently map to TS Cassette and Other Cassette exons than to Constitutive exons (p < 2.2 × 10^−16^, chi-square). In other words, Constitutive exons more often mapped to Pfam protein domains (Figure 2A). However, when a TS Cassette exon mapped to a protein domain, it was more common that the segment mapped to a whole domain compared to Other Cassette or Constitutive exons (5%; 72/1,426 exons; Table S1A). A handful of protein domains were enriched in TS exons (p < 10^−2^, Fisher's exact test; Table S1B). These domains have a role in binding linear peptide motifs on other protein partners (e.g., SH3 domain), PTMs on protein partners (e.g., ubiquitin interaction motif), nucleic acids (e.g., basic helix-loop-helix domain), or small molecule ligands or ions (e.g., calcium-binding EGF domain). Domains associated with TS exons also have a role in regulation and in mediating interactions with other protein partners (e.g., SCAN domain with a role in transcription regulation; Table S1A). This indicates that TS exons encoding complete domains may have a role in influencing different types of protein interactions. However, the low propensity of protein domains to be included in TS Cassette exons (72 of 1,426 exons) suggests that most protein segments encoded by these exons do not have a well-defined tertiary structure and that their primary mechanism of function is not via structured regions.

### Tissue-Specific Protein Segments Frequently Contain Disordered Regions

Prediction of disordered regions using the IUPred program revealed that alternatively spliced exons were more frequently associated with disordered regions than were constitutive exons: 31% (442/1,426) and 22% (2,970/13,755) of segments encoded by TS and Other Cassette exons, respectively, had more than half of their residues in intrinsically disordered regions, compared to 16% (21,400/137,046) of the Constitutive exons (Figure 2B; see figure for p values). Protein segments encoded by TS Cassette exons had the highest proportion of disordered regions. While this trend is seen in all individual tissues, some tissues (e.g., brain) more often contained disordered regions in TS segments than others (e.g., Colon; Table S1D). These observations were robust to changes in cutoff values used to define intrinsically disordered regions (Figure S1B) and the disorder prediction method employed. We further examined whether protein disorder is a specific feature of the segments encoded by TS exons, or whether it is a general feature of full-length proteins that contain these segments. A comparison of the fraction of exons that encode disordered segments revealed that disorder is more frequently associated with alternatively spliced TS exons (31%; 442/1,426) compared to the non-TS exons (21%; 3,543/16,850) from the same genes (p < 2.2 × 10^−16^, chi-square). Therefore, the observed high fraction of disorder is not solely a generic property of the proteins encoded by genes with such exons but tends to be specific to TS Cassette exons.

### Tissue-Specific Protein Segments Are Enriched in Predicted Binding Motifs

In order to assess whether TS Cassette exons are enriched in binding motifs that can be recognized by other proteins, we investigated if they encoded peptide interaction motifs that could be bound by globular domains (Davey et al., 2012; Van Roey et al., 2012). Using the ANCHOR program, which predicts regions that undergo disorder-to-order transition upon binding, we observed that the fraction of segments that overlapped a predicted binding motif was significantly higher for segments encoded by TS Cassette exons than for Other Cassette or Constitutive exons (Figure 2C; see figure for p values): 44% (634/1,426) compared to 30% (or 4,180/13,755) and 26% (or 35,185/137,046), respectively. These observations were independent of differences in exon lengths among the different exon categories (Table S1E). Among TS segments with a long intrinsically disordered region (349 segments; defined as stretches of at least 30 amino acids assigned as “disordered” with a maximum of three “ordered” amino acids), 89% contained a predicted binding motif. In a majority of the cases, there were two or more binding motifs per intrinsically disordered region. Taken together, these results suggest that TS segments frequently contain disordered regions that embed peptide motifs and are likely to be involved in mediating interactions.

### Tissue-Specific Protein Segments Are Enriched in PTM Sites

We obtained PTM annotations from the UniProt/Swiss-Prot Database and considered only exons for which representative isoforms had an exact match in the Swiss-Prot Database. The majority (94%) of PTM sites encoded by TS exons correspond to phosphorylation sites, and a majority (74%) were present in disordered regions. We observed that TS Cassette exons encoded PTM sites significantly more often than did other exons (Figure 2D; see figure for p values). The fraction of segments with at least one PTM site was significantly higher for the TS exons (13% or 119/917) compared to Other Cassette exons (7% or 477/6,746) or Constitutive exons (8% or 6,795/86,198). The reported observations were not biased by the size of exon data sets or by the distribution of exon lengths in different data sets (Table S1E). In a further attempt to eliminate annotation bias toward the phosphorylation sites, we analyzed phosphorylation sites identified in a single unbiased large-scale experiment (Gnad et al., 2007) and found the results to be in agreement with the above observations (Table S1F). These results show that segments encoded by TS Cassette exons are significantly enriched in PTM sites.

### Tissue-Specific Exons Are Conserved at the DNA and Protein Levels

To analyze the evolution of TS exons, we first investigated genomic alignments of human and mouse orthologs and found that DNA sequences corresponding to human TS exons were significantly more conserved than the other exon categories (Figure S2A). At the protein level, TS segments were highly conserved in general, but the overall level of conservation was comparable to the other exon types (Figure S2B). For all exons, the ratio of nonsynonymous (Ka) to synonymous (Ks) substitution rate was much lower than 1, suggesting overall purifying selection. However, the Ka/Ks ratio was significantly higher for TS exons compared to the other exon types (Figure S2C). A comparison of the Ka and Ks values revealed that this was not due to increased Ka, but due to a significantly smaller Ks (Figures S2D and S2E). In other words, this pattern emerges from selection against mutations in synonymous sites rather than neutral evolution or a weak positive selection in TS exons. This finding is consistent with previous observations on alternatively spliced exons and is a signature of purifying selection for functional elements at the nucleic acid level (Xing and Lee, 2005a, 2006b). Such functional elements may be splicing enhancer or exclusion elements, specific RNA secondary structures, or sites for RNA binding proteins that may impact on mRNA function, localization, or stability (Chamary et al., 2006; Xing and Lee, 2006a).

### Disordered Regions and Binding Motifs in Tissue-Specific Segments Are Conserved at the Protein Level

To analyze the importance of protein functional elements, we investigated evolutionary patterns in the different exon types. The regions encoding disordered segments and predicted binding motifs were both more conserved for the TS exons compared to the other exon types at the DNA level (Figures S2F and S2G). At the protein level, both disordered regions and binding motifs in TS exons were more conserved than those in Other Cassette exons (Figures 2E and 2F). Furthermore, within a TS segment, amino acids in binding motifs were more conserved than the rest of the segment (Figure 2G). These observations suggest selection for functional elements both at the DNA and the protein level within disordered regions of TS exons. Further investigation of mutation patterns in regions that map to predicted binding motifs and the other parts of the exon revealed that mutations in synonymous positions appear to be tolerated in regions that map to predicted binding motifs despite their higher evolutionary conservation at the protein level (Figures S2H–S2J). This suggests that the predicted binding motifs at the protein level are indeed under purifying selection. It should be stressed that since neither disorder prediction nor binding site prediction methods take into account evolutionary information, the observed higher conservation of these regions at the protein level likely indicates their functional importance.

### Genes with Tissue-Specific Exons Tend to Occupy Central Positions in Protein Interaction Networks

**Figure 3:**
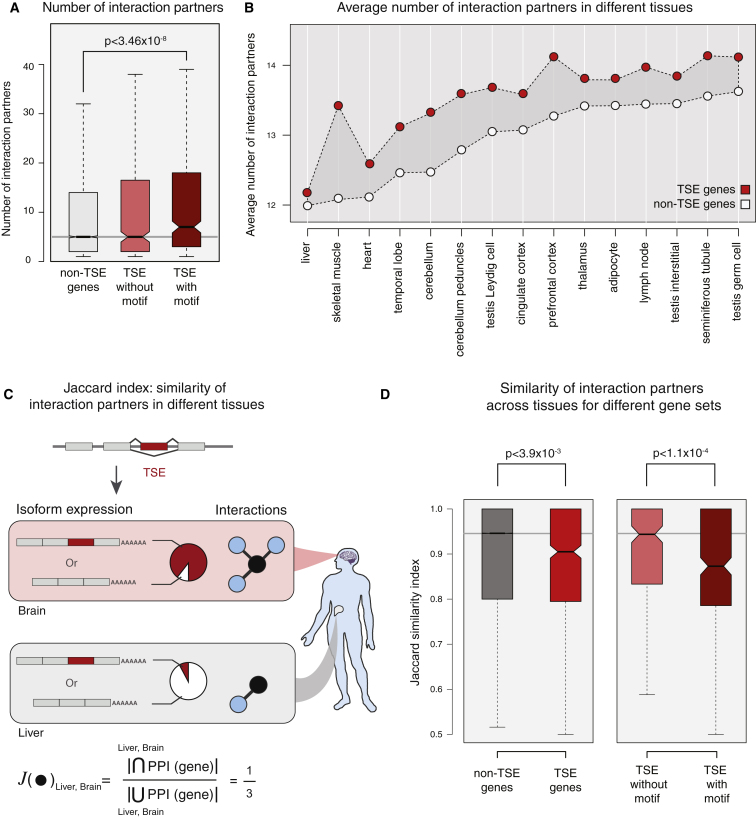
Genes with Tissue-Specific Exons Play an Important Role in Protein Interaction Networks (A) Distribution of the number of interaction partners for genes with a TS exon (TSE genes; with and without predicted binding motif) and for all other genes (non-TSE genes). See Figure 2 legend for the description of a box plot. (B) Average number of interaction partners for the TSE (red circles) and non-TSE (gray circles) genes in different tissues. The average and median number of interactions for TSE genes was greater than for non-TSE genes in each of the tissues (p < 5 × 10^−2^; Mann-Whitney), except for liver and testis interstitial cells. (C) Principle of the Jaccard similarity index. The score for a gene varies between 0 and 1, where 0 indicates that no interactions are maintained and 1 indicates that all interactions are maintained between a pair of tissues. Hence, the smaller the number is, the more tissue specific are its interactions. (D) Distribution of Jaccard similarity indices for TSE and non-TSE genes in the tissues included in this study. Gray horizontal line in all box plots indicates median value for the non-TSE genes. Statistical significance was assessed using Mann-Whitney test. See also Figure S3 and Table S2.

We investigated the significance of genes with TS Cassette exons (TSE genes) by mapping them onto the protein interaction network. Using the integrated human protein-protein interaction (PPI) network (Bossi and Lehner, 2009), we found that the TSE genes have on average more interaction partners than genes that do not contain TS Cassette exons (non-TSE genes) (p < 1.43 × 10^−5^, Mann-Whitney). Further, a subset of TSE genes that contain predicted disordered binding motifs tend to have more interaction partners than the other TSE genes (Figure 3A). This is consistent with our observation that genes with TS Cassette exons are enriched for roles in interaction and binding (Table S2). The importance of a protein in a PPI network can also be quantified by computing its centrality in the network. Using several metrics such as betweenness, closeness, page rank, and Kleinberg's hub score (Supplemental Experimental Procedures), we found that genes containing TS exons (particularly the subset with predicted binding motif) have on average a higher centrality than the non-TSE genes in the PPI network (Figure S3A). This suggests that perturbing their function might impact a larger number of proteins and that the inclusion or exclusion of exons in a tissue-specific manner has the potential to rewire interactions in the protein interaction network.

### Genes with Tissue-Specific Exons Tend to Have Distinct Interaction Partners in the Respective Tissue

By investigating the set of human tissue-specific protein interaction networks in the individual tissues included in the analysis (Bossi and Lehner, 2009), we observed that the TSE genes again had on average more interaction partners than non-TSE genes (Figure 3B). We then investigated how PPIs are maintained in pairs of tissues, in which a TS Cassette exon is either included or excluded (Supplemental Experimental Procedures). For this, we calculated the Jaccard similarity index, i.e., the proportion of interactions that are maintained in a pair of tissues out of all possible interactions seen in both tissues (Figure 3C). Jaccard similarity index ranges from 0 to 1, where 0 indicates that no interaction is maintained and 1 indicates that all interactions are maintained in the two tissues. We found that the mean Jaccard similarity index is significantly lower for TSE genes compared to the non-TSE genes in the investigated tissues. This suggests the presence of a significantly higher fraction of tissue-specific protein interactions for TSE genes, compared to non-TSE genes that are expressed in the same pairs of tissue (Figure 3D, left; and Figure S3B). Further, the subset of TSE genes that contain a predicted disordered binding motif tend to have even lower average Jaccard similarity index, suggesting a significantly higher fraction of tissue-specific protein interactions mediated by these genes (Figure 3D, right). These observations support the idea that differential inclusion of such segments can mediate distinct protein interactions in these tissues. A Monte Carlo simulation confirmed that the reported observations are unlikely to be observed by chance (Figure S3C). In addition, a systematic analysis of the variability in interaction partners across the different tissues by using a measure of information entropy revealed that TSE genes (particularly those with predicted binding motif) had significantly more distinct interaction partners compared to the non-TSE genes (Figure S3D and Supplemental Experimental Procedures). Whether this pattern emerges due to the alternative inclusion of a TS cassette exon or due to the distinct expression profile of interaction partners (or both) may be addressable in the future.

### Genes with Tissue-Specific Exons Are Enriched in Developmental and Disease Genes

The high connectivity of the genes with TS exons in the protein interaction network suggests that mutating them is likely to manifest in a disease primarily because such genes are likely to be pleiotropic. To investigate this, we used (1) experimentally annotated phenotype data for mouse genes with human orthologs and (2) information about cancer-related human genes. This showed that genes whose human orthologs have tissue-specific isoforms (p < 1.2 × 10^−8^, chi-square) are enriched in genes that cause embryonic lethality in mice when deleted (Mouse Genome Database). Additionally, genes that have been causally implicated in cancer (Cancer Gene Census Database) and genes found to be somatically mutated in different cancer types (COSMIC Database) were both enriched to encode TS exons (p < 6.2 × 10^−2^ and p < 3.2 × 10^−6^, respectively, chi-square; Figure S4, Table S5A). These observations suggest that genes with TS exons are enriched in development and disease genes.

### Genes with Tissue-Specific Exons Are Enriched in Signaling Pathways

Since developmental and cancer genes are often involved in signaling pathways, we investigated whether TSE genes are enriched in specific functions pertaining to signaling. Using the DAVID functional annotation server, we observed that genes with TS exons were enriched in molecular function GO terms such as protein binding, nucleic acid binding, and kinase activity (Table S2). Since alternative inclusion of functional protein segments could be a mechanism for adapting the same gene to function in different pathways, we investigated whether TSE genes were also known to be involved in multiple signaling pathways. For this, we used data from the SignaLink Database, which currently contains 60 human multipathway genes. We found that seven genes with TS Cassette exons were part of two or more curated signaling pathways. Although this appears to be a small number, TSE genes are nevertheless enriched in multipathway genes (p < 3 × 10^−2^, Fisher's exact test). These genes belong to evolutionarily conserved and developmentally important pathways such as the EGF/MAPK, Hedgehog, WNT/wingless, TGF-β, and JAK/STAT pathways (Table 1). These results indicate that genes encoding TS Cassette exons are enriched to occur in signaling pathways.

### Literature Evidence Supports the Role of Tissue-Specific Protein Segments in Mediating Molecular Interactions

Collectively, these results suggest that TS splicing can affect molecular interactions. To find further experimental support for this hypothesis, we performed an extensive search for instances where the TS exons overlapped with experimentally verified binding motifs using the STRING, UniProtKB/Swiss-Prot, and ELM databases and literature data. We identified 35 regions where the functional importance of the TS segment is enabled by either inter- or intramolecular interactions (Table 2, Figures S3A and S6). In all these cases, an independent experiment in the literature has corroborated the role of the interacting residues, thus relating the presence of a TS segment with the protein's ability to recognize its interaction partners. Consistent with our observations, the function, in most cases, involves modulation of different signaling pathways.

**Figure 4:**
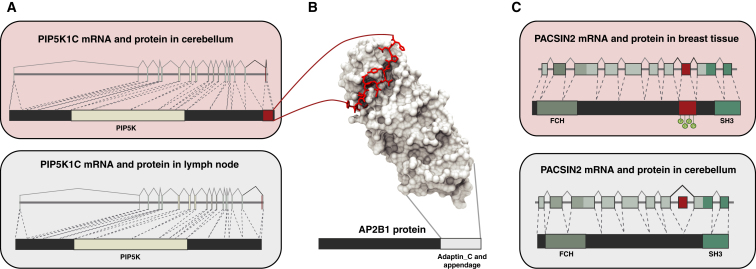
Examples of Tissue-Specific Exons that Can Affect Protein Interactions Transcripts (above) and the encoded protein sequences (below) are shown for tissues (box). Protein domains (colored regions) and tissue-specific segments (red) are shown. (A) The *PIP5K1C* gene shows differential inclusion of a TS exon between cerebellum and lymph node. This segment undergoes disorder-to-order transition when interacting with the clathrin adaptor AP-2 (AP2B1). The exclusion of this segment is likely to abolish the interaction. (B) Protein structure (3H1Z), containing interacting segments from these two proteins. (C) *PACSIN2* shows differential inclusion of a TS exon between cerebellum and breast tissue. All four annotated phosphorylation sites are inside the TS exon. Thus, the exclusion is likely to affect regulation via phosphorylation. See also Table S3.

Furthermore, an investigation of the available structural data in the Protein Interfaces, Surfaces, and Assemblies (PISA) Database identified 138 TS segments that had at least one amino acid involved in forming an interaction interface with other proteins, DNA, or RNA in the complex (Table S3B; 60 TS segments map to likely biologically relevant interface, including homo-oligomers). An example includes the *PIP5K1C* kinase, which shows a significant difference in inclusion levels of a disordered TS segment between cerebellum and lymph node (Figure 4A). The interaction with the Adaptor protein 2 (*AP2*), which is crucial for vesicle formation, is mediated by a peptide motif within the disordered segment (Figure 4B). This binding motif is, however, tissue-specifically excluded in the isoform expressed in the lymph node, and this likely abolishes the interaction between the two proteins.

Finally, we also identified several instances where a PTM site was included within a region encoded by TS exon. An example includes the *PACSIN2* gene, which is involved in vesicle formation and transport. This gene can be phosphorylated by protein kinase C, thereby regulating its interactions with other proteins. All four annotated phosphorylation sites map within a TS exon that is skipped in cerebellum but included in the breast tissue. This suggests that alternative inclusion is likely to abolish certain interactions of *PACSIN2* in the cerebellum by preventing the regulation through protein kinase C (Figure 4C). Another 23 TS segments with experimentally validated phosphorylation sites are provided in Table S3C. Thus, supporting the predicted trends, there are several annotated and experimentally validated examples, which illustrate how splicing of TS segment can impact protein interactions, PTM, and function.

## Discussion

While some studies have shown that alternative splicing has a general tendency to avoid protein domains and include disordered regions (Hegyi et al., 2011; Kriventseva et al., 2003; Pentony and Jones, 2010; Romero et al., 2006), other studies have shown that a vast majority of alternatively spliced isoforms could potentially cause significant alterations in regions of the protein structural core (Tress et al., 2007; Yura et al., 2006). Our integrated analysis, which compares constitutive, tissue-specific, and other alternative exons, shows that (1) tissue-specifically spliced exons are significantly enriched to encode disordered regions that embed protein-binding motifs, (2) constitutive exons more often map to protein domains than other exons, and (3) other alternative cassette exons show a trend that is in between the two.

Previous studies investigating the conservation of DNA sequences reported that alternatively spliced exons evolve faster than constitutive exons (Chen et al., 2012; Modrek and Lee, 2003). A study on a smaller set of TS exons obtained using splicing microarrays in mouse, however, showed that these exons were highly conserved at the DNA sequence level (Xing and Lee, 2005b). Consistent with this, we note that human TS exons display higher conservation and decreased mutation rates in synonymous sites. The latter suggests selection for functional elements at the level of nucleic acids that may additionally constrain protein sequence evolution. Such nucleotide sequence elements in TS exons can influence mRNA splicing, localization, or stability (Chamary et al., 2006; Parmley et al., 2006; Witten and Ule, 2011; Xing and Lee, 2005a, 2006a).

Given that tissue-specific protein segments are enriched in disordered regions, they are expected to evolve rapidly due to the absence of structural constraints (Bellay et al., 2011; Brown et al., 2011; Romero et al., 2006). In contrast, we find that disordered regions encoded by TS exons are more conserved than those encoded by other alternative exons. We also observe that the amino acids within predicted peptide-binding motifs in tissue-specific segments were more conserved compared to other amino acids in the same segment. Moreover, regions mapping to predicted binding motifs appear to tolerate synonymous mutations to a certain degree, despite higher conservation at the protein level. This suggests that in addition to the above-mentioned nucleotide sequence elements that influence mRNA function, the predicted protein-binding motifs embedded in disordered regions appear to serve as additional constraints on the evolution of TS exons.

**Figure 5:**
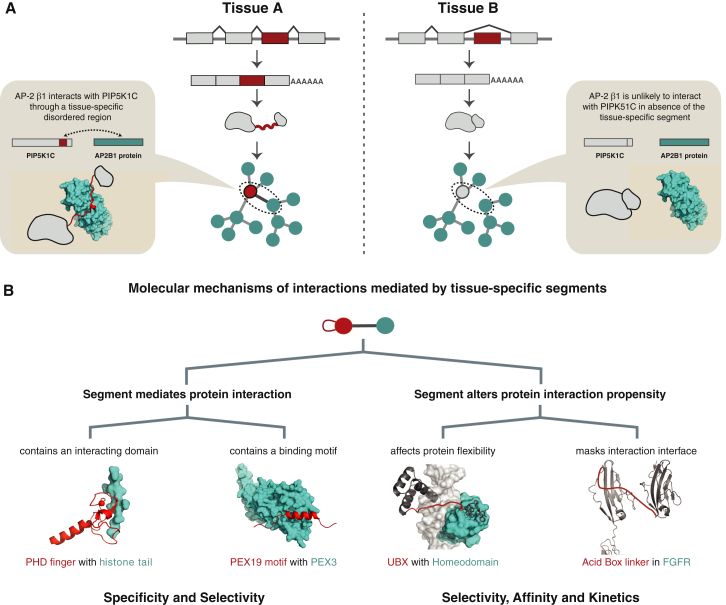
Alternative Inclusion of Tissue-Specific Exons Can Rewire Interaction Networks and Modulate Protein Interactions (A) When tissue-specific splicing gives rise to isoforms that differ in the presence of interacting protein segments, this can result in the rewiring of an interaction network in the respective tissues. This is illustrated with the *PIP5K1C* kinase gene that has an exon with different inclusion levels in cerebellum and lymph mode. The exon encodes a binding motif (red cartoon representation), which mediates the interaction with the AP-2β appendage domain (cyan surface representation). (B) Molecular mechanisms by which a tissue-specific segment can rewire or fine-tune protein interactions. Segments in red are encoded by TS exons; other regions encoded by the same protein are colored dark gray. Interaction partners are colored in cyan. Segments encoded by TS exons can include a region that directly interacts with other proteins, DNA, RNA, or ligands, or indirectly affect the protein's binding properties (e.g., affinity, kinetics, and selectivity). TS segments that are involved in interactions are less frequently domains and more often disordered regions that embed peptide-binding motifs. An example for the former is a TS segment encoding the complete PF21A PHD zinc finger domain that recognizes nonmethylated lysine 4 of histone H3 (2PUY). An example for the latter is the disordered segment encoded by a TS exon of PEX19, which becomes ordered upon binding to PEX3 (3MK4). An example of a splicing event that affects linker length, thereby altering its affinity to bind DNA, is the fly *UBX* gene (1B8I; disordered linker, red line; and DNA, light gray surface). An example of where the spliced exon encodes an intramolecular interaction motif that competes or masks an interaction interface is the acid box region of the FGFR kinases (model based on 2CKN and 1YRY; acidic linker, red line). See also Table S4.

Through an analysis of expression and protein interaction network data, we observed that genes encoding tissue-specific protein isoforms tend to (1) have more interaction partners on average compared to the other genes and (2) occupy central positions in the protein interaction network. More importantly, genes that contain TS exons tend to make interactions that are distinct in different tissues compared to genes that do not contain TS exons. These observations suggest that tissue-specific splicing may mediate new interactions in a tissue-specific manner through the alternative inclusion of disordered segments that contain binding motifs (Figure 5A). In this way, tissue-specific splicing could rewire molecular interactions and change the topology of signaling and regulatory pathways by modulating the inclusion of binding motifs or interaction domains in a tissue-specific manner.

Collectively, these observations raise the question of the molecular mechanisms by which tissue-specific segments influence protein interactions. Based on the observed characteristics of TS segments and the literature examples, we delineate genome-scale molecular principles by which such segments can influence protein function and their interaction networks.

### Tissue-Specific Protein Segments Can Confer Specificity to an Interaction

Several TS protein segments that encode binding regions have already been implicated in mediating interactions in the literature (see Table 2 and Tables S3A–S3C). Furthermore, the functional role of the predicted binding motifs is supported by our observation that they are more conserved than other residues in TS exons. Even though domains are generally depleted in these segments, those that are present in TS segments are also involved in mediating molecular interactions and are devoid of functional roles such as enzyme catalysis. This suggests that by alternatively including segments that encode protein domains or disordered binding motifs, tissue-specific segments may achieve specificity in protein interactions (Figure 5B, left). Specific examples include the putative tissue-specifically spliced Zn finger domain of the PHD finger protein 21A that interacts with unmethylated lysine 4 of histone H3 and the tissue-specifically spliced disordered binding motif in peroxisomal biogenesis factor 19 protein that interacts with Pex3, important for peroxisome biogenesis (Table 2; Figure 5B, left).

Consistently, genes with TS segments encoding binding motifs tend to have interaction partners that are distinct in different tissues. Thus, tissue-specific splicing can potentially lead to the recruitment of the same molecular function, often carried out by structured domains encoded by constitutive exons, to different contexts by mediating new molecular interactions through the disordered segment (Tables S1C and S5B). For instance, TS isoforms of the same kinase gene could mediate recruitment of different proteins as substrates in a tissue-specific manner (see Table S4B for candidate kinase genes from our analysis). In a similar manner, the differential inclusion of disordered segments that encode PTM sites can make the same protein a potential substrate of different signaling enzymes (e.g., kinases) in a tissue-specific manner.

### Tissue-Specific Segments Can Influence Selectivity by Affecting the Affinity and Kinetics of an Interaction

We also observe a subset of TS exons to encode disordered segments that do not contain binding motifs. Such segments can still affect interactions with other partner molecules by affecting the linker length between domains or binding motifs (e.g., Ca^2+^/calmodulin-dependent protein kinase in Table 2). They can also act as allosteric regulators of interface formation (Figure 5B, middle right). For instance, a change in the length of a disordered region can increase the conformational entropy and hence interfere with an interaction elsewhere on the protein (Fuxreiter et al., 2011; Hilser and Thompson, 2007). In addition, the net charge of the disordered segments, which can be further modulated by PTMs, can act as affinity tuners of an interaction (Keren et al., 2010; Vuzman and Levy, 2012). In this context, our observation that TS segments show an enrichment to encode PTM sites suggests that their alternative inclusion might also alter the affinity and kinetics of protein interactions.

Literature examples in which isoforms with variable length in the disordered region can cause disease or are important for development include the human and fly transcription factors (TFs), Wilms' tumor gene *WT1* (Laity et al., 2000), and Ultrabithorax (Liu et al., 2008), respectively. In both cases, splicing does not affect the DNA binding domain itself but changes the length of disordered segment, leading to altered DNA sequence specificity and affinity. Notably, we found several human TFs with disordered TS segments (see Table S4C). It is possible that these TS segments will be governed by similar molecular principles and could hence modulate interaction properties.

### Tissue-Specific Segments Can Affect Response Kinetics and Cellular Decisions

Motifs in disordered segments can also compete for an interaction interface on the same protein through self-interaction (autoinhibitory peptides). An example is the alternatively spliced acid box (AB) region of several members of the fibroblast growth factor receptor family of kinases (see Table 2; Figure 5B, right). These segments are intrinsically disordered and play a key role in the autoinhibition of the kinase, thereby regulating signaling in the absence of the ligand. Similarly, the simultaneous expression of multiple isoforms in the same tissue can result in competition for similar interaction partners or altered kinetics upon signal input such as an ultrasensitive response (Buchler and Louis, 2008). For instance, a shorter isoform of *p53* can affect cellular differentiation by titrating full-length *p53* and competing for the same DNA sequence (Ungewitter and Scrable, 2010). The same phenomenon could well apply to tissue-specific isoforms. Given that (1) genes with TS exons have more interaction partners and occupy central position in interaction networks and (2) the flanking domains of disordered TS exons (Table S1C) are often involved in binding other proteins (e.g., bromodomain), nucleic acids (e.g., Myb DNA binding and RRM) or small molecules (e.g., FYVE), the differential inclusion of TS segments can result in isoforms that compete for similar interaction partners. In this manner, tissue-specifically spliced segments can enable and fine-tune molecular interactions, cellular outcomes, or cell-fate decisions by influencing parameters such as response kinetics across different cell types and tissues.

### Implications for Disease and Evolution

From the perspective of disease and drug development, de novo mutations in TS exons can result in altered interaction properties and may lead to cell-type-specific diseases such as cancer (Shan et al., 2012). While harder to achieve, one could nevertheless aim to develop isoform-specific drugs that may have fewer side effects compared to drugs that target constitutively spliced regions (Spitali and Aartsma-Rus, 2012). From an evolutionary perspective, the prevalence of binding motifs in tissue-specific segments may serve as a remarkably simple mechanism for the formation of novel interactions in protein networks (Mosca et al., 2012). The findings also suggest how “intrinsically less evolvable” proteins such as developmentally important TFs with conserved DNA binding domains can explore new functional landscapes through differential inclusion of such segments, for instance, by facilitating recruitment of other TFs or chromatin-modifying enzymes through disordered binding motifs. Importantly, given the growing evidence for species-specific (Pan et al., 2005) and sex-specific (Blekhman et al., 2010) splicing of genes, it is likely that such exons could contribute to the emergence of organism- and sex-specific interaction networks.

In conclusion, one of the prevalent outcomes of splicing of TS exons appears to be modulation of protein-binding properties through the alternative inclusion of disordered segments that contain binding motifs. In this context, our characterized list of TS exons (Table S4A) can guide large-scale proteomics studies in different tissues and delineate the molecular principle of tissue-specific interactions involving TSE genes on a case-by-case basis. While we discuss how such regions can rewire or tune protein interactions and influence cellular decisions, the same principles could also influence protein-DNA, protein-RNA, and protein-ligand interaction networks on a genomic scale. In this manner, tissue-specific splicing might contribute to the functional versatility of proteins and shape the interaction networks in different tissues in multicellular organisms. This plasticity may lead to the emergence of novel phenotypes and increased complexity during organismal evolution.

## Experimental Procedures

### Exon Data Set

TS Cassette exons were composed by mapping cassette exons with differential tissue inclusion levels, as reported by Wang and coworkers (Wang et al., 2008), to Ensembl protein-coding exons (release 54; http://www.ensembl.org/). The tissues include adipose, brain, breast, cerebellum, colon, heart, liver, lymph node, skeletal muscle, and testes as well as BT474, HME, MB435, MCF7, and T47D cell lines. The set of Other Cassette exons were identified using the following criteria: the exon (1) was alternatively present in at least two transcripts, (2) was not mutually exclusive with an adjacent exon, and (3) did not overlap with TS Cassette exons, and (4) there was another RNA isoform that also contained different exons upstream and downstream of the exon of interest. This was necessary to avoid cases of alternative start and termination sites. Constitutive exons were identified as those that were present in all transcript products with unchanged boundaries. See the Supplemental Experimental Procedures for details.

### Analysis of Structural Properties of the Encoded Protein Segments

Protein domains were predicted using the Pfam software (release 25; http://pfam.sanger.ac.uk/). Intrinsically disordered regions were predicted with both the IUPred (short mode; http://iupred.enzim.hu/) and VSL2B (http://www.dabi.temple.edu/disprot/readmeVSL2.htm) software. For the structural analysis, TS segments extracted from the Ensembl proteins were first mapped to protein sequences in the UniProt (http://www.uniprot.org/) and SwissProt Knowledgebase (http://www.expasy.org/). Regions that were in PDB (http://www.pdb.org/) were obtained for the UniProt/SwissProt KB canonical sequences.

### Analysis of Functional Sites in the Encoded Protein Segments

ANCHOR (http://anchor.enzim.hu/) software was used to predict binding motifs. PTM sites were obtained from the UniProt/SwissProt KB. Only UniProt entries that were identical to the representative Ensembl proteins were used. Structures of proteins with TS segments in complex with other protein and or DNA and RNA were obtained from the PDB and PISA databases (http://pdbe.org/pisa), and residues at the interface were identified.

### Analysis of Evolutionary Conservation of the Segments

Identifiers and sequences of one-to-one human-mouse orthologs were obtained via Ensembl. When more than 90% of the residues in a TS segment were covered in the alignment, the segment was considered as present in the mouse protein. The alignments were obtained using the Needleman-Wunsch algorithm in the EMBOSS package. See the Supplemental Experimental Procedures for more details.

### Analysis of Protein-Protein Interaction Networks

Human tissue-specific PPIs were obtained from the literature (Bossi and Lehner, 2009). Network analyses were restricted to the consensus interactome and to tissues that were present in both studies. The number of interaction partners (i.e., degree), and measures of centrality such as betweenness, closeness centrality, Kleinberg's hub score, and page rank were calculated with the iGraph library in R. The fraction of protein interaction partners that were maintained between the two tissues was calculated using the Jaccard similarity index (Supplemental Experimental Procedures). TSE genes with binding motifs were defined as those that contained at least ten amino acids in the predicted motif.

### Statistical Significance

All statistical tests employed in this analysis were performed using the R statistical package.

## Figures and Tables

**Table 1:** Genes with Tissue-Specific Exons that Participate in More Than One Signaling Pathway

HGNC Gene Name	Gene Description from ENSEMBL	Tissues or Cell Lines with Differential Exon Inclusion	Signaling Pathways
PRKACA	Protein kinase, cAMP-dependant, catalytic, alpha	Cerebellum and MB435	EGF/MAPK^NC^, Hedgehog^C^, WNT/Wingless^C^
GSK3B	Glycogen synthase kinase 3 beta	MCF7 and cerebellum	EGF/MAPK^C, NC^, Hedgehog^C^, TGF-β^C^, WNT/Wingless^C^
AXIN1	Axin 1	Cerebellum and T47D, cerebellum and MCF7	EGF/MAPK^NC^, TGF-β^C,NC^, WNT/Wingless^C^
DAB2	Disabled homolog 2, mitogen-responsive phosphoprotein	Breast and colon, cerebellum and heart	TGF-β^NC^, WNT/Wingless^NC^
TGFBR2	Transforming growth factor, beta receptor II	Heart and testes	EGF/MAPK^C^, TGF-β^C^
IRAK1	Interleukin-1 receptor-associated kinase 1	Cerebellum and skeletal muscle	EGF/MAPK^NC^, JAK/STAT^NC^
DAXX	Death-domain-associated protein	Breast and cerebellum, breast and heart	EGF/MAPK^NC^, TGF-β^NC^

Multipathway genes were obtained from the SignaLink Database (http://signalink.org/); core and noncore pathway genes are denoted by ^C^ and ^NC^, respectively. See also Figure S4 and Table S5.

**Table 2:** Examples Where Tissue-Specific Segments Overlap with Experimentally Verified Interaction Sites

Protein Name (Tissue-Specifically Spliced Region)	UniProt Accession (Annotated Binding Region)	Binding Partners (PMID)	Description of Interaction
**Intermolecular Interactions**			

*Annexin VII* (1–18)	ANXA7_HUMAN (5–20)	Sorcin (9268363)	Annexin VII functions in membrane fusion events. The N-terminal repeat region of Annexin VII (residues 1–31) is required for the calcium-dependent interaction with the EF-hand protein sorcin.
*Axin-1* (1–292)	AXIN1_HUMAN (20–29)	Tankyrase (22307604)	The tumor suppressor Axin-1 regulates the Wnt/β catenin signaling pathways. Turnover of Axin-1 is controlled by tankyrase. Axin-1 contains two Tankyrase binding motifs (residues 18–30 and 60–80). The putative TS isoform of Axin-1 lacks both of these motifs.
*PHD finger protein 21A* (484–535)	PF21A_HUMAN (488–535)	Histone H3 (17687328)	PF21A is a component of the BRAF-HDAC complex and mediates transcriptional repression of neuron specific genes in nonneuronal cells. The putative TS isoform of PF21A lacks a PHD zinc finger domain that recognizes unmethylated lysine 4 of histone H3.
*Cdc42-interacting protein 4* (329–384)	CIP4_HUMAN (293–537)	CDC42, TC10 (9210375, 12242347, 19387844)	The F-BAR protein CIP-4 has an important role in endocytosis. The second coiled-coil domain of CIP4 (residues 332–425) is required for interaction with the GTPases CDC-42 and TC-10.
*Integrin-linked kinase* (30–85)	ILK_HUMAN (33–139)	LIMS1 [PINCH] (19074270)	Integrin-linked kinase has been implicated in cell adhesion, growth factor, and Wnt/β catenin signaling pathways. The adaptor protein PINCH is overexpressed in several types of cancers. The ankyrin repeats of ILK (residues 2–154) interact with the first LIM domain of PINCH.
*Mannan-binding lectin serine peptidase 1* (2–79)	MASP1_HUMAN (20–278)	FCN2, MBL2 (18596036)	MASP-1 is a component of the mannan binding lectin pathway of the complement system. The putative TS exon of MASP-1 encodes a signal peptide (1–19) and part of a Cub-1 domain (20–138). The latter is involved in calcium-dependent homodimerization of MASP-1 and in the interaction with Mannan Binding Lectin 2 as well as Ficolin.
*Negative regulator of ubiquitin-like proteins 1* (417–465)	NUB1_HUMAN (427–474)	NEDD8 (12816948)	Alternative splicing modulates the role of Nub-1 as an important negative regulator of the NEDD8 conjugation system. A longer Nub-1 isoform (Nub1L) possesses a third UBA motif required for Nedd8 interaction.
*Nuclear transcription factor, X-box binding 1* (9–344)	NFX1_HUMAN (9–26)	PABPC1, PABC4 (17267499)	The interaction of the N-terminal PAM2 motif (9–26) of NFX1 with the cytoplasmic poly(A) binding proteins PABPC1 and PABC4 is important for the role of NFX1 in regulation of telomerase activity (hTERT transcription).
*Perixomal biogenesis factor 19* (24–60)	PEX19_HUMAN (1–56)	PEX3 (21102411)	The interaction of Pex19 and Pex3 is critical for peroxisome formation and the posttranslational targeting of peroxisomal membrane proteins. Pex3 interacts with residues 17–32 of Pex19, which are disordered in the unbound state.
*Phosphatidylinositol-4-phosphate 5-kinase, type I, gamma* (641–668)	PI51C_HUMAN (641–668)	TLN2, AP-2μ, AP-2β (12422219, 19903820)	The predominant brain splice variant of PtdInsPKI gamma contains a short C-terminal recognition motif. Interaction of this motif with the FERM domain of Talin targets and activates the kinase for focal adhesion assembly during cell migration and synaptic vesicle recycling at nerve terminals. Moreover, it has been found that the same C-terminal region of PtdInsPKI interacts with the appendage domain of AP-2β and the signal-sorting domain of AP-2μ during clathrin-mediated endocytosis.

**Intramolecular Interactions**			

*Calcium/Calmodulin-dependent kinase II* (329–339, 316–340)	KCC2A_HUMAN, KCC2B_HUMAN (autoinhibition)	Intramolecular autoinhibition of CamKII (21884935)	CamKII functions in neuronal signaling, and the interaction of the N-terminal kinase domain with the C-terminal Hub domain is important for autoinhibition of the enzyme. The linker length between these two domains differs between the paralogues, providing a mechanism to fine-tune the strength of the interaction and autoinhibition. The TS exons encoding for same linker region are also alternatively spliced.
*Fibroblast growth factor receptor 2* (56–144)	FGFR2_HUMAN (autoinhibition)	Intramolecular autoinhibition of FGFR2 (22244757)	Uncontrolled fibroblast growth factor (FGF) signaling has been implicated in human diseases. The acidic linker region between the first and second immunoglobulin domains (D1 and D2) interacts with D2 and maintains an autoinhibitory state of FGFR. Certain isoforms lack D1 and/or the acidic linker region, leading to increased capacity of FGF signaling.

**Signal Peptides**			

*Complement C2* (16–85)	CO2_HUMAN (1–20)	not annotated (7852336, 19237749)	Complement C2 is a part of the classical pathway of the complement system, and deficiencies of this protein are associated with autoimmune diseases. A short TS splice isoform of Complement C2 retains an intracellular localization, as it lacks the N-terminal signal peptide required for secretion. Moreover, it lacks a CCP domain that is involved in the interaction with C4b.
*RPA interacting protein* (16–85)	RIP_HUMAN (164–180)	not annotated (16135809)	RPA functions in DNA replication, repair, and recombination. RIP interacts with RPA and determines thereby the subcellular localization of RPA. A region of RIP (164–180), required for its cytoplasmic retention, has been shown to be encoded by an alternatively spliced exon.

Definitions of protein regions that mediate an interaction were derived from the UniProt Database, and the functional description are summarized from the literature. PubMed identifiers (PMIDs) are provided as reference for interactions.

## Supplemental Information

Document S1. Figure S1, Figure S2, Figure S3, Figure S4, Table S1, Table S2, Table S3, Table S4, Table S5, Supplemental Experimental Procedures, and Supplemental References
