# Supplementary material for: Tissue-Specific Splicing of Disordered Segments that Embed Binding Motifs Rewires Protein Interaction Networks
Source: Mol Cell. 2012 Jun 29;46(6):871–83. doi: 10.1016/j.molcel.2012.05.039 (PMC3437557; doi:10.1016/j.molcel.2012.05.039)
Supplement: Document S1. Figure S1, Figure S2, Figure S3, Figure S4, Table S1, Table S2, Table S3, Table S4, Table S5, Supplemental Experimental Procedures, and Supplemental References [file mmc1.pdf]

Molecular Cell, Volume *46*

Supplemental Information

Tissue-Specific Splicing of Disordered Segments that Embed Binding Motifs Rewires Protein Interaction Networks

Marija Buljan, Guilhem Chalancon, Sebastian Eustermann, Gunter Wagner, Monika Fuxreiter, Alex Bateman, and M. Madan Babu

CONTENTS:

SUPPLEMENTAL INFORMATION

Supplemental Figures S1-S4

Supplemental Tables S1-S5

SUPPLEMENTAL EXPERIMENTAL PROCEDURES

SUPPLEMENTAL REFERENCES

INVENTORY OF MAIN DISPLAY AND SUPPLEMENTAL DATA

Figure 1: Classification of exons and flowchart for the characterization of the encoded protein segments

Figure S1: *Events that result in the alternative inclusion of exonic sequences (**A**). Fractions of exons that encode disordered protein segments, according to different thresholds for disorder definition (**B**)*

Figure 2: Tissue-specific segments are enriched in disordered binding motifs and are highly conserved

Figure S2: *Evolutionary conservation of whole exons at the level of nucleic acids (**A**) and amino acids (**B**). dN/dS (**C**), dN (**D**) and dS (**E**) for the different sets of exons. Evolutionary conservation of disordered region (**F**) and binding motif (**G**) at the nucleic acid level. dN/dS (**H**), dN (**I**) and dS (**J**) for binding regions and other regions in TS exons.*
Table S1A: *List of protein domains encoded by tissue-specific exons*
Table S1B: *Enriched protein domains encoded by tissue-specific exons*
Table S1C: *Neighbouring domains in proteins with tissue-specific disordered segments*
Table S1D: *Percentage of splice events affecting disordered segments in the different tissues*
Table S1E: *Control calculations for exon datasets of the same size and the same median exon length*
Table S1F: *Distribution of phosphosites among the three datasets of exons*

Figure 3: Genes with tissue-specific exons play an important role in protein interaction networks

Figure S3: *Centrality measures for the different gene sets (**A**), Averaged Jaccard similarity index for all pairs of tissues (**B**), Monte-Carlo simulation results for the different gene sets (**C**), Entropy calculation for quantifying variation in interaction partner across different tissues for the different groups of genes (**D**).*
Table S2: *Functional enrichment of genes with tissue-specific exons, with motifs and long disorder*

Figure 4: Examples of tissue-specific exons that can affect protein interactions

Table S3A: *Further examples of TS exons mapping to interaction interface from literature.*
Table S3B: *Genes with tissue-specific exons that map to a protein interaction interfaces*
Table S3C: *Genes with tissue-specific exons that contain PTM sites*

Figure 5: Alternative inclusion of tissue-specific protein segments can rewire and modulate protein interactions

Table S4A: *Genes with TS exons that encode predicted interaction motifs in disordered segments*
Table S4B: *List of kinases that contain a tissue-specific segment with predicted interaction motifs but no detectable Pfam protein domains*
Table S4C: *List of transcription factors that contain a tissue-specific segment with predicted disordered regions but no detectable Pfam protein domains*

Table 1: Genes with tissue-specific exons that are involved in multiple signaling pathways

Figure S4: *Genes with tissue-specific exons are enriched in embryonic lethal and disease genes*
Table S5A: *Genes associated with embryonic lethality and genes that were found mutated in cancer are enriched in the genes with tissue-specific exons*
Table S5B: *List of domains that are most frequently encoded by genes that contain tissue-specific exons but are not encoded by the tissue-specific exons themselves*

Table 2: Literature examples of tissue specific regions that overlap with experimentally verified interaction sites

SUPPLEMENTAL DATA

Supplemental Figures

A

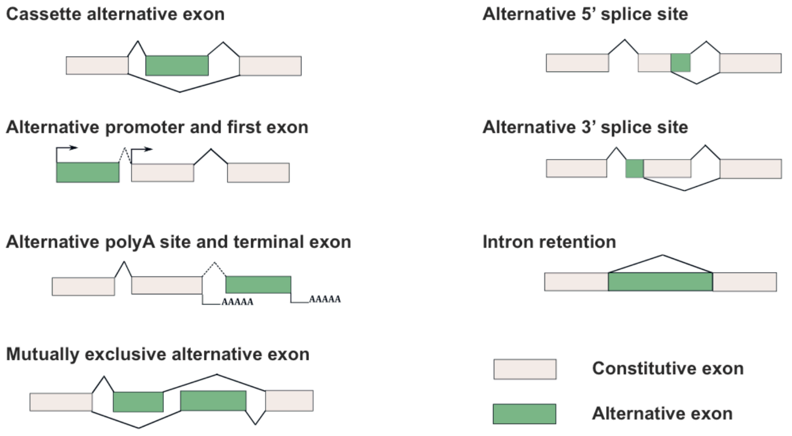

B

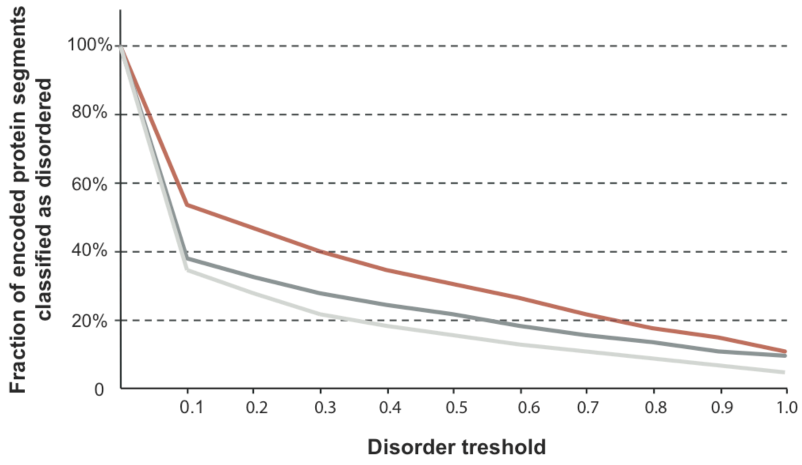

Figure S1, related to main figure 1: Different types of alternative exon inclusion and prevalence of disorder in tissue-specific cassette exons. (A) Events that result in the alternative inclusion of exonic sequences. Exonic sequences that are alternatively present in the mature transcripts are colored green. Cassette exons are exons that are alternatively included in, or excluded from, the mature transcripts with their whole length and their inclusion is decided during splicing. Alternative inclusion of terminal exons occurs through the usage of alternative start and termination sites. Mutually exclusive exons (MXEs) are also differentially included with their whole length, but inclusion of one MXE implies exclusion of the other. When there is more than one 5' or 3' splice site, exon borders can differ depending which of these sites is recognized during splicing. Intron retention is the phenomenon where an intron is read out as a part of an exon and included in the mature transcript. Isoforms resulting from the processes on the left differ in the presence of whole exons and those resulting from splice events on the right differ in length of the spliced exon. In our study, we analysed only cassette alternative exons, and distinguished between tissue-specific and other cassette exons. (**B**) **Fraction of exons encoding disordered segment according to different thresholds for disorder definition**. The lines show the fraction of exons, which encode disordered protein segments, in the studied sets of exons: Tissue-specific Cassette exons (red line), Other Cassette exons (dark grey line) and Constitutive exons (light grey line). The X-axis shows different thresholds for a fraction of disorder content in a protein segment that were used to define disordered segment. The Y-axis shows a fraction of exons in each set that were encoding disordered segments defined by using different thresholds. For example, the fraction of residues classified as disordered according to the IUPred is on average 0.4 for known disordered proteins in the Disprot database (Sickmeier et al., 2007). By using this value as a threshold, we observed a similar trend to those presented in **Figure 2B**: TS Cassette, Other Cassette and Constitutive exons encoded 35%, 25% and 19% disordered segments.

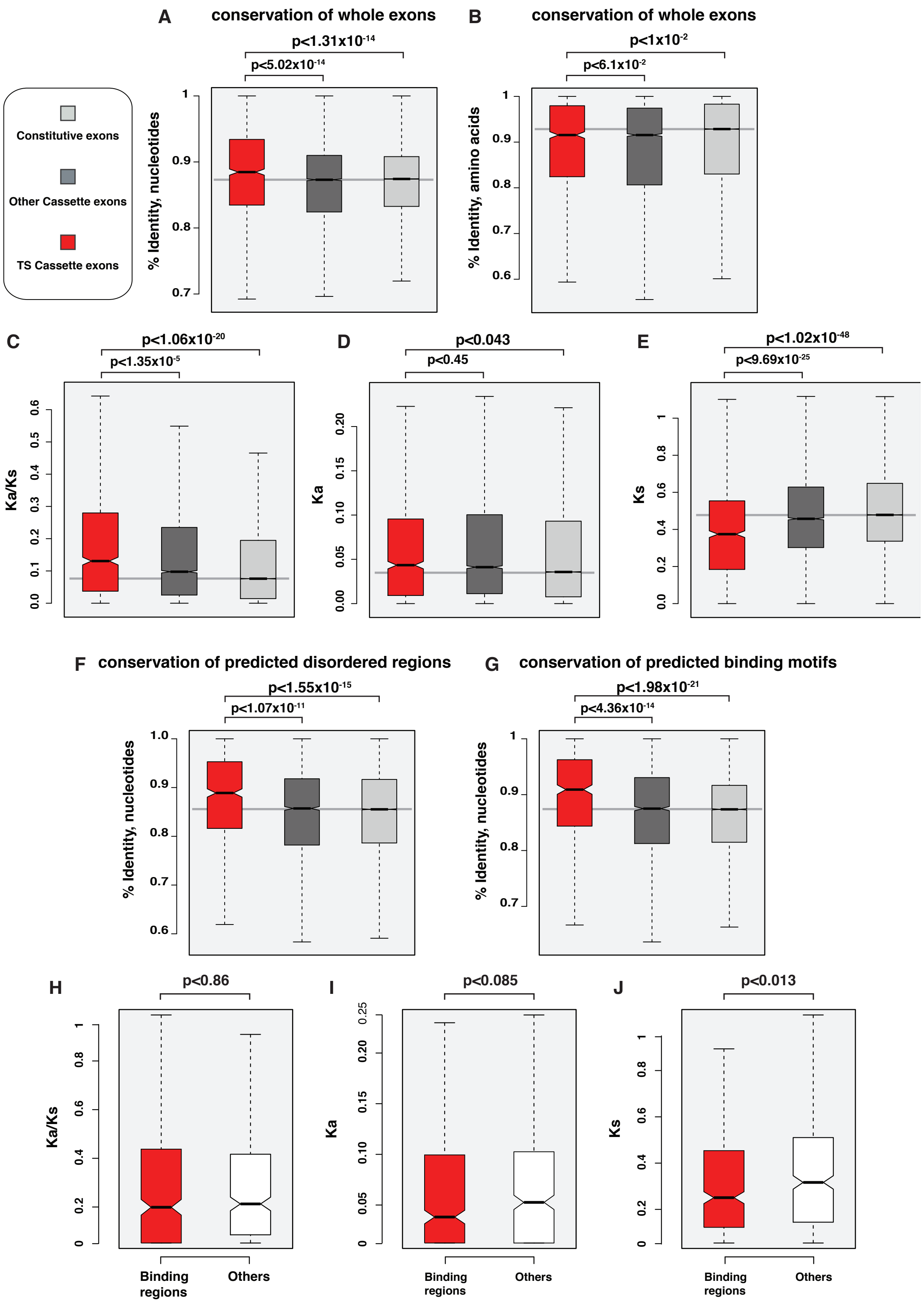
A
conservation of whole exons
p<1.31x10-14
p<5.02x10-14
% Identity, nucleotides
B
conservation of whole exons
p<1x10-2
p<6.1x10-2
% Identity, amino acids
Constitutive exons
Other Cassette exons
TS Cassette exons
C
p<1.06x10-20
p<1.35x10-5
Ka/Ks
D
p<0.043
p<0.45
Ka
E
p<1.02x10-48
p<9.69x10-25
Ks
F
conservation of predicted disordered regions
p<1.55x10-15
p<1.07x10-11
% Identity, nucleotides
G
conservation of predicted binding motifs
p<1.98x10-21
p<4.36x10-14
% Identity, nucleotides
H
p<0.86
Ka/Ks
I
p<0.085
Ka
J
p<0.013
Ks
Binding regions
Others

Figure S2, related to main figure 2: Patterns in the evolution of entire exons and features within exons. See main Figure 2 legend for the description of a boxplot. Conservation of whole exons at the nucleic acid (**A**) and protein level (**B**). Distribution of Ka/Ks ratio (**C**), Ka (**D**) and Ks (**E**) values for the different classes of exons. Ks values are significantly lower for the TS exons compared to other exon types suggesting presence of selection pressures for functional elements at the nucleotide level. Conservation of predicted disordered regions (**F**) and binding motifs (**G**) for the different exon types at the DNA level. Distribution of Ka/Ks ratio (**H**), Ka (**I**) and Ks (**J**) values for regions that map to predicted binding motifs and other regions within the same TS exons. The Ks values are lower but marginally significant for regions mapping to binding motifs compared to the other regions (for an indication, please see statistical significance in panel E). This suggests that synonymous substitutions are not very strongly selected against and may be tolerated in regions mapping to peptide binding motifs.

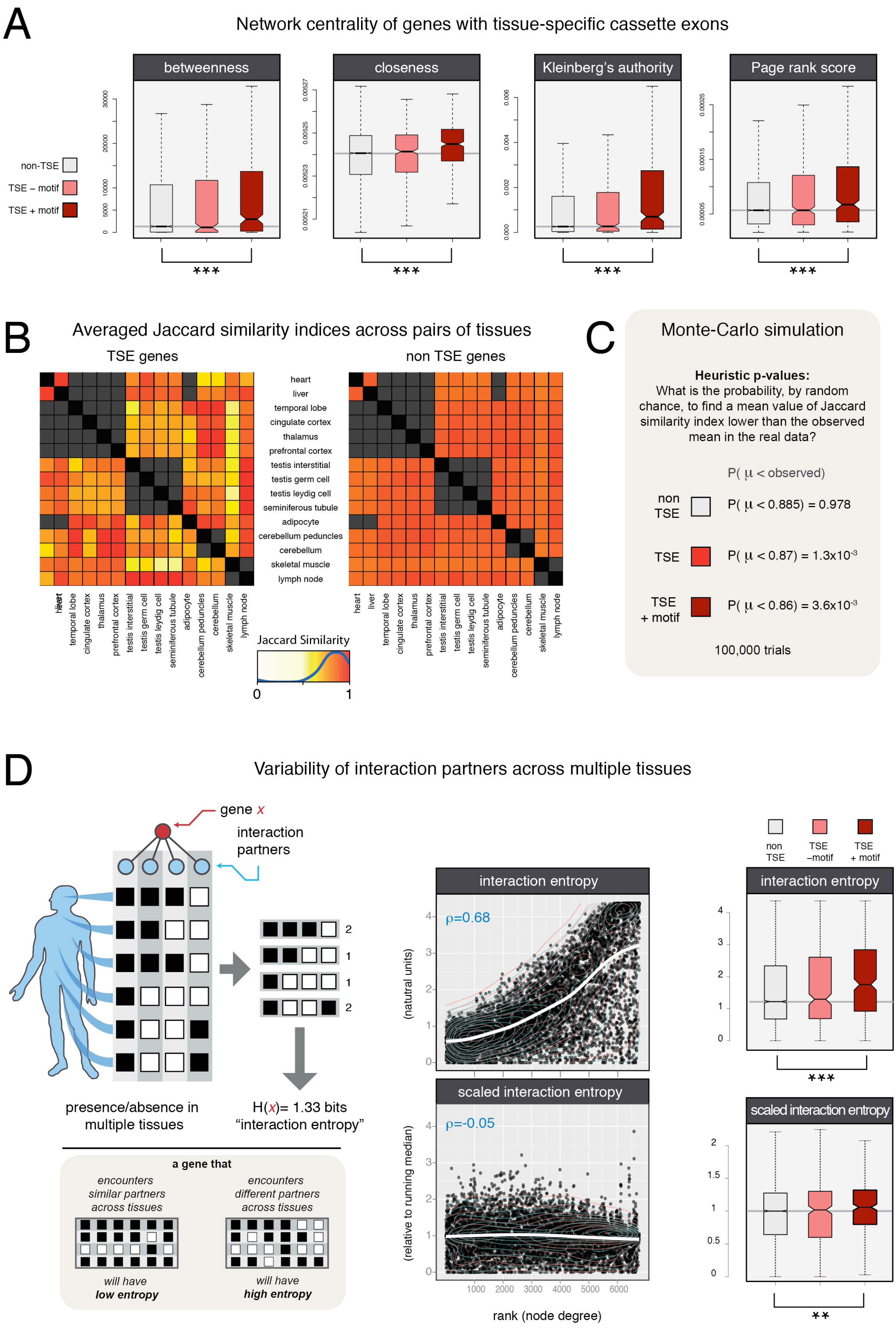

A
Network centrality of genes with tissue-specific cassette exons
betweenness
closeness
Kleinberg's authority
Page rank score
non-TSE
TSE – motif
TSE + motif
***
B
Averaged Jaccard similarity indices across pairs of tissues
TSE genes
non TSE genes
heart
liver
temporal lobe
cingulate cortex
thalamus
prefrontal cortex
testis interstitial
testis germ cell
testis leydig cell
seminiferous tubule
adipocyte
cerebellum peduncles
cerebellum
skeletal muscle
lymph node
Jaccard Similarity
0
1
C
Monte-Carlo simulation
Heuristic p-values:
What is the probability, by random chance, to find a mean value of Jaccard similarity index lower than the observed mean in the real data?
P(μ < observed)
non TSE
P(μ < 0.885) = 0.978
TSE
P(μ < 0.87) = 1.3x10-3
TSE + motif
P(μ < 0.86) = 3.6x10-3
100,000 trials
D
Variability of interaction partners across multiple tissues
gene x
interaction partners
presence/absence in multiple tissues
H(x)= 1.33 bits
"interaction entropy"
a gene that
encounters similar partners across tissues
encounters different partners across tissues
will have low entropy
will have high entropy
interaction entropy
ρ=0.68
(natural units)
scaled interaction entropy
ρ=-0.05
(relative to running median)
rank (node degree)
non TSE
TSE –motif
TSE + motif
***
**

Figure S3, related to main figure 3: Importance of genes with tissue-specific exons in interaction networks. See main Figure 2 legend for the description of a boxplot. (**A**) Measurements of network centrality for non-TSE genes and TSE genes with or without predicted binding motif. In each case, TSE genes with motif have significantly higher centrality score than non-TSE genes (all $p<1.43 \times 10^{-3}$, Mann-Whitney test). (**B**) Matrix representation of averaged Jaccard similarity indices across all pairs of tissues, ranging from 0 (white) to 1 (red). (**C**) Results of the Monte-Carlo simulation. (**D**) Left panel: principle of the calculation and interpretation of interaction entropy. Middle: Quantification of interaction entropy, ordered by increasing node degree. Spearman rank correlation coefficients are given for raw values as well as scaled values, showing that the dependency between interaction entropy and node degree is removed by the scaling. Right: TSE genes with predicted binding motifs have significantly higher interaction entropy compared to non-TSE genes (difference in median = 0.30 bits, $p<5.48 \times 10^{-8}$, Mann-Whitney test). When removing the contribution of node degree to information entropy (scaled interaction entropy), TSE genes with interaction motifs remain significantly higher (+5.2%, $p<2.3 \times 10^{-3}$, Mann-Whitney test) compared to non-TSE genes.

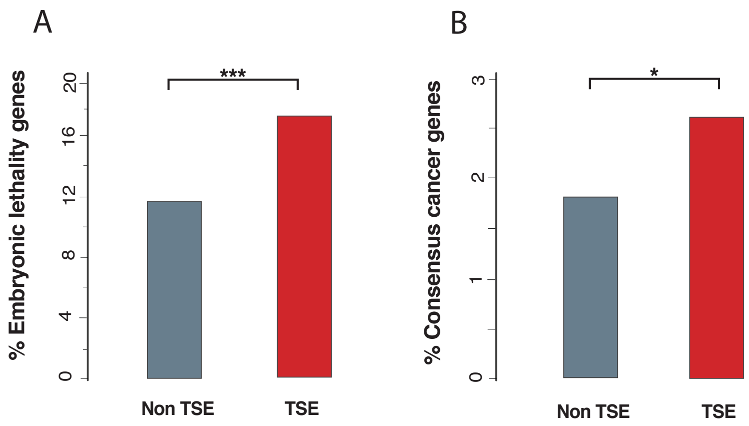

Figure S4, related to main table 1: Genes with tissue-specific exons are enriched in embryonic lethal and disease genes. Fraction of genes with Tissue-specific Cassette exons (TSE columns in the figure) that are associated with embryonic lethality (**A**) or cancer (**B**) is significantly higher than the fraction of all other human genes associated with these (Non TSE columns in the figure). *** denotes $p<1.2x10^{-8}$ and * denotes $p<6.2x10^{-2}$; Chi-square test.

Supplemental Tables

Table S1A,B,C,D,E,F, related to main figure 2

Table S1A: Protein domains encoded by Tissue-specific Cassette exons. Pfam protein domains and families, which were encoded by at least one Tissue-specific exon, i.e. at least 90% of a domain length was encoded by the exon, are listed. The number of different Tissue-specific exons that encoded the same domain is shown in the column titled No.

Domain identifier	Domain	No	Representative transcripts encoding a TS region with the domain
PF07645	EGF_CA	7	ENST00000354476, ENST00000301873, ENST00000295761, ENST00000308370, ENST00000331782, ENST00000308370, ENST00000308874
PF00096	zf-C2H2	4	ENST00000173785, ENST00000232014, ENST00000235372, ENST00000396801
PF07686	V-set	3	ENST00000356709, ENST00000360141, ENST00000400376
PF00018	SH3_1	3	ENST00000379446, ENST00000216733, ENST00000233154
PF00041	fn3	3	ENST00000350763, ENST00000354785, ENST00000359947
PF00010	HLH	2	ENST00000262965, ENST00000344749
PF02809	UIM	2	ENST00000270460, ENST00000268933
PF07679	I-set	2	ENST00000404537, ENST00000374737
PF00084	Sushi	2	ENST00000375510, ENST00000375472
PF02023	SCAN	2	ENST00000396852, ENST00000330501
PF00090	TSP_1	2	ENST00000373658, ENST00000258613
PF12796	Ank_2	2	ENST00000264607, ENST00000375018
PF01352	KRAB	2	ENST00000337673, ENST00000219069
PF12937	F-box-like	1	ENST00000355619
PF00357	Integrin_alpha	1	ENST00000375221
PF00651	BTB	1	ENST00000397753
PF00397	WW	1	ENST00000282441
PF07654	C1-set	1	ENST00000367580
PF00581	Rhodanese	1	ENST00000366899
PF00856	SET	1	ENST00000358065
PF00619	CARD	1	ENST00000268605
PF01335	DED	1	ENST00000336034
PF00487	FA_desaturase	1	ENST00000305631
PF01846	FF	1	ENST00000345122
PF12799	LRR_4	1	ENST00000369452
PF00057	Ldl_recept_a	1	ENST00000315571
PF00014	Kunitz_BPTI	1	ENST00000263574
PF12872	OST_LOTUS	1	ENST00000355295
PF00685	Sulfotransfer_1	1	ENST00000304842
PF00645	zf-PARP	1	ENST00000378526
PF02351	GDNF	1	ENST00000306793
PF00418	Tubulin-binding	1	ENST00000344290
PF00035	dsrm	1	ENST00000389862

PF12736	CABIT	1	ENST00000373921
PF01928	CYTH	1	ENST00000404535
PF00628	PHD	1	ENST00000257821
PF05033	Pre-SET	1	ENST00000358065
PF00615	RGS	1	ENST00000262320
PF12874	zf-met	1	ENST00000219782
PF11409	SARA	1	ENST00000371591
PF04146	YTH	1	ENST00000373812
PF01754	zf-A20	1	ENST00000380828
PF05624	LSR	1	ENST00000361790
PF00560	LRR_1	1	ENST00000369452
PF00092	VWA	1	ENST00000338660
PF00612	IQ	1	ENST00000367409
PF10558	MTP18	1	ENST00000407550
PF11608	Limkain-b1	1	ENST00000396368
PF00135	COesterase	1	ENST00000264381
PF00071	Ras	1	ENST00000345122
PF01129	ART	1	ENST00000355810
PF08916	Phe_ZIP	1	ENST00000359285

Table S1B: Protein domains enriched in tissue-specific segments. Pfam domains that were more frequently encoded by TS Cassette exons than by Other Cassette or by Constitutive exons are listed together with the p-values (Fisher's exact test for a comparison with the two sets of exons).

Domain name	Pfam identifier	Number of TS exons with domain	P-value for a comparison with Other Cassette exons	P-value for a comparison with Constitutive exons
Calcium-binding EGF domain	PF07645	7	$2.0x10^{-3}$	$6.4x10^{-3}$
SH3 domain	PF00018	3	$6.7x10^{-3}$	$6.2x10^{-3}$
Basic helix-loop-helix domain	PF00010	2	$8.4x10^{-3}$	$2.4x10^{-2}$
Ubiquitin interaction motif	PF02809	2	$8.4x10^{-3}$	$1.8x10^{-3}$

Table S1C: Neighbouring domains in proteins with TS disordered segments. Domains that do not overlap TS segments but are present in the same protein (in at least three proteins) with a disordered TS segment are listed. The number of proteins that contain both a TS segment that is not disordered and the same domain elsewhere in a protein is also shown.

Domain identifier	% Proteins with domain & disordered TS segment	Proteins with domain & disordered TS segment	Proteins with domain & TS segment that is not disordered	Domain name
CL0257	100%	3	0	Mec-17
PF00641	100%	3	0	zf-RanBP
PF12202	100%	3	0	OSR1_C
PF00105	100%	3	0	zf-C4
PF02209	100%	4	0	VHP
PF07529	100%	3	0	HSA
CL0271	100%	3	0	F-box-like
PF07815	100%	3	0	Abi_HHR
PF00104	100%	3	0	Hormone_recep
CL0214	83%	5	1	UBA
PF00017	80%	4	1	SH2
PF00098	75%	3	1	zf-CCHC
CL0357	75%	3	1	FHA
PF00791	75%	3	1	ZU5
CL0145	75%	6	2	BAR
PF01412	75%	3	1	ArfGap
CL0266	69%	20	9	PID
PF00439	67%	6	3	Bromodomain
CL0049	67%	6	3	MBT
CL0007	67%	6	3	KH_1
PF00373	67%	4	2	FERM_M
CL0072	62%	8	5	UBX
CL0010	61%	20	13	SH3_1
CL0031	60%	3	2	DSPc
CL0137	60%	3	2	Hydrolase
PF00621	60%	3	2	RhoGEF
PF00611	60%	3	2	FCH
CL0114	57%	4	3	CHDNT
CL0466	56%	10	8	PDZ
CL0390	55%	6	5	FYVE
CL0016	53%	17	15	Pkinase
CL0023	52%	17	16	NTPase_1
CL0020	50%	12	12	Leuk-A4-hydro_C
CL0465	50%	9	9	Ank_2
CL0188	45%	5	6	CAMSAP_CH
CL0409	45%	5	6	RhoGAP
CL0221	43%	12	16	RRM_1
CL0159	40%	12	18	IL6Ra-bind
CL0202	38%	3	5	PITH
CL0011	37%	10	17	C1-set
PF00412	36%	4	7	LIM
CL0229	33%	3	6	zf-C3HC4
CL0361	32%	6	13	zf-C2H2
CL0186	29%	9	22	Kelch_1
CL0063	23%	3	10	Methyltransf_18
CL0123	21%	3	11	Myb_DNA-binding

Table S1D: Percentage of splice events affecting disordered segments in the different tissues. Out of all tissue-specific exons that are alternatively included in a given tissue, a fraction of exons that encode disordered alternative segments (i.e. at least half of the residues in the segment were in disordered regions) is shown for each tissue.

Tissue	% of splice events affecting disordered segment	Splice events affecting disordered protein segment	Other splice events in the same tissue
**Brain**	39%	56	86
**Heart**	36%	30	54
**Skeletal muscle**	33%	50	101
**Testes**	32%	67	140
**Adipose**	32%	53	112
**Cerebellum**	31%	163	370
**Lymph node**	30%	68	160
**Breast**	28%	74	189
**Colon**	27%	39	103
**Liver**	24%	13	41

Table S1E: Control calculations for exon datasets of the same size and with the same median exon length. Subsets of Other Cassette and Constitutive exons of the same size and of the same median exon length as Tissue-specific Cassette exons were composed through filtering and random sampling of the original exon datasets. For a reliable comparison, 1,000 random subsets of these exons were created. Distribution of the fractions of exons in these subsets that were (i) intrinsically disordered, (ii) contained a PTM site or (iii) contained a binding motif was noted. Shapiro-Wilk test in R was used to test whether the distributions of the tested values deviated from the normal distribution (p-values smaller than would 0.05 indicate a significant deviation). Cumulative distribution function in R was used to compare the averages and standard deviations of values of the examined properties in the subsets of Other Cassette and Constitutive exons to the fraction of exons with these properties in the set of Tissue-specific exons.

Analysis	Set of exons	Number of exons in a set	Average fraction of the examined feature	S.D. of the feature	Shapiro-Wilk p-value	Cumulative distribution function p-value
**Disorder (IUPred)**	TS Cassette	1,426	31%	/	/	/
	Other Cassette	1,426	17%	0.9%	P<0.5	$P<3.4\times10^{-56}$
	Constitutive	1,426	13%	0.9%	P<0.13	$P<7.2\times10^{-89}$
**Binding motifs (ANCHOR)**	TS Cassette	1,426	44%	/	/	/
	Other Cassette	1,426	32%	1.1%	P<0.13	$P<6.4\times10^{-25}$
	Constitutive	1,426	26%	1.2%	P<0.8	$P<4.4\times10^{-54}$
**PTM sites**	TS Cassette	917	13%	/	/	/
	Other Cassette	917	8%	0.9%	P<0.9	$P<1.2\times10^{-8}$
	Constitutive	917	8%	0.9%	P<0.5	$P<2.1\times10^{-7}$

Table S1F: Distribution of phosphosites among the three datasets of exons. The phosphosite dataset used in the analysis was previously identified in a single mass spectrometry study. Fractions of exons in the three different exon datasets, which encoded at least one phosphosite, were compared (column $Fraction_{+}$). The N_{+} column shows the number of exons with at least one phosphosite and the N_{-} shows the number of exons without a phosphosite. p-values show statistical significance of the comparisons between the Tissue-specific exons on one side and Other Cassette or Constitutive exons on the other side. Statistical significance of the differences in distribution of phosphosites was tested with the Chi-square test in R.

Set of exons	N_{+}	N_{-}	N_{total}	$Fraction_{+}$	p-value
TS Cassette	28	1,188	1,216	2.3%	/
Other Cassette	56	10,294	10,350	4%	$P<2.7x10^{-11}$
Constitutive	559	112,781	113,340	5%	$P<2.2x10^{-16}$

Table S2, related to main figure 3

Table S2: Functional enrichment of genes with tissue-specific exons, and of the subsets of the genes with disordered tissue-specific segments and with binding motifs inside tissue-specific segments. Molecular function Gene Ontology (GO) terms that the genes with Tissue-specific Cassette exons were enriched in ($p<5x10^{-2}$), when compared to all other human genes, are listed. EASE p-values represent modified Fisher exact p-values. The 'Benjamini p-value' column shows corrected p-values after applying the Benjamini-Hochberg correction for multiple tests. To obtain these values, gene annotations and implemented statistical tests in the DAVID database were used. P-values are given for (a) a subset of TSE genes with disordered tissue-specific segments (b) for the subset of TSE genes with binding motif (defined as at least 5 amino acids in the predicted motif) and (c) all genes with tissue-specific segments.

TSE genes with disordered regions			
GO.ID	Term	P-value	Benjamini
GO:0005515	protein binding	3.98E-17	1.92E-14
GO:0005488	binding	1.10E-11	2.66E-09
GO:0008092	cytoskeletal protein binding	6.08E-11	9.77E-09
GO:0003779	actin binding	4.49E-10	5.41E-08
GO:0008134	transcription factor binding	6.45E-08	6.22E-06
GO:0017124	SH3 domain binding	9.70E-07	7.79E-05
GO:0030695	GTPase regulator activity	6.38E-05	4.38E-03
GO:0060589	nucleoside-triphosphatase regulator activity	8.71E-05	5.23E-03
GO:0003712	transcription cofactor activity	1.17E-04	6.24E-03
GO:0003713	transcription coactivator activity	2.21E-04	1.06E-02
GO:0005083	small GTPase regulator activity	2.93E-04	1.28E-02
GO:0019904	protein domain specific binding	2.99E-04	1.19E-02
GO:0016563	transcription activator activity	5.71E-04	2.10E-02
GO:0005096	GTPase activator activity	9.60E-04	3.25E-02
TSE genes with binding motif			
GO.ID	Term	P-value	Benjamini
GO:0005515	protein binding	1.83E-21	8.87E-19
GO:0005488	binding	1.47E-15	3.51E-13
GO:0008092	cytoskeletal protein binding	5.08E-11	8.23E-09
GO:0003779	actin binding	1.42E-09	1.73E-07
GO:0008134	transcription factor binding	3.37E-09	3.27E-07
GO:0003676	nucleic acid binding	4.22E-07	3.42E-05
GO:0017124	SH3 domain binding	6.07E-07	4.21E-05
GO:0030695	GTPase regulator activity	6.13E-06	3.72E-04
GO:0019899	enzyme binding	8.56E-06	4.62E-04
GO:0060589	nucleoside-triphosphatase regulator activity	9.44E-06	4.58E-04
GO:0017016	Ras GTPase binding	1.12E-05	4.96E-04
GO:0019904	protein domain specific binding	1.16E-05	4.70E-04

GO:0030528	transcription regulator activity	1.22E-05	4.56E-04
GO:0051020	GTPase binding	1.48E-05	5.15E-04
GO:0003723	RNA binding	2.30E-05	7.44E-04
GO:0031267	small GTPase binding	3.28E-05	9.95E-04
GO:0005083	small GTPase regulator activity	4.64E-05	1.33E-03
GO:0003677	DNA binding	1.29E-04	3.49E-03
GO:0003712	transcription cofactor activity	1.41E-04	3.59E-03
GO:0005096	GTPase activator activity	6.51E-04	1.57E-02
GO:0016563	transcription activator activity	8.27E-04	1.90E-02
GO:0008307	structural constituent of muscle	1.20E-03	2.62E-02
GO:0003713	transcription coactivator activity	1.37E-03	2.85E-02
GO:0003700	transcription factor activity	2.04E-03	4.05E-02
All TSE genes			
GO.ID	Term	P-value	Benjamini
GO:0005515	protein binding	1.15E-18	1.19E-15
GO:0008092	cytoskeletal protein binding	3.15E-10	1.63E-07
GO:0005488	binding	1.05E-08	3.63E-06
GO:0003779	actin binding	8.63E-08	2.23E-05
GO:0008134	transcription factor binding	1.09E-06	2.26E-04
GO:0017124	SH3 domain binding	1.20E-06	2.07E-04
GO:0000166	nucleotide binding	9.36E-06	1.38E-03
GO:0003723	RNA binding	2.79E-05	3.61E-03
GO:0016740	transferase activity	7.19E-05	8.24E-03
GO:0016772	transferase activity, transferring phosphorus-containing groups	9.15E-05	9.44E-03
GO:0008093	cytoskeletal adaptor activity	1.04E-04	9.70E-03
GO:0019904	protein domain specific binding	1.07E-04	9.18E-03
GO:0005516	calmodulin binding	1.61E-04	1.27E-02
GO:0003713	transcription coactivator activity	2.38E-04	1.75E-02
GO:0003824	catalytic activity	3.37E-04	2.30E-02
GO:0003712	transcription cofactor activity	4.00E-04	2.55E-02
GO:0008757	S-adenosylmethionine-dependent methyltransferase activity	4.32E-04	2.60E-02
GO:0016563	transcription activator activity	7.86E-04	4.42E-02

Table S3A,B,C, related to main figure 4

Table S3A: Further examples where tissue specific segments overlap with experimentally verified interaction sites. Definitions of functionally important regions were derived from the UniProt database, which contained experimental binding data from protein-protein interaction databases and the literature.

Gene	UniProt Protein accession	TS region	UniProt Binding region	UniProt Binding partner	Reference (PubMed ID)
**Inter-molecular Interactions**					
Cyclin-dependent kinase inhibitor 3	CDKN3_HUMAN	4-30	1-34	CDK2	15530371, 11463386
Death domain-associated protein 6 (DAXX)	DAXX_HUMAN	1-69	1-160	SUMO-1	21383010
Dynamin 1-like (isoform 2)	DNM1L_HUMAN isoform 2	533-558	448-632	GSK3B	9731200
Protein 4.1N	E41L1_HUMAN	484-495	483-541	Actin	11050113
Leucine-rich repeat flighless-interacting protein 2	LRRF2_HUMAN	346-369	1-370	DVL3	15677333
Myotubularin related protein 12	MTMRC_HUMAN	505-558	449-558	MTM1	11504939
Nuclear autoantigenic sperm protein (histone-binding)	NASP_HUMAN	137-475	469-512	Histone H4A, H3A, H3B, H1	18782834
Nuclear receptor coactivator 6	NCOA6_HUMAN	972-1964	1-1057	LXXLL motif 2, CREBBP	11997499
Nucleoporin like 2	NUPL2_HUMAN	41-116	94-170	HIV-1 VPR	12228227
Poly(A) polymerase alpha	PAPOA_HUMAN	690-714	677-745	NUDT21	21102410
Nucleoporin like 2	NUPL2_HUMAN	41-116	94-170	HIV-1 VPR	12228227
Rab interacting lysosomal protein	RILP_HUMAN	315-342	272-333	RAB7	15933719
Similar to ribonucleic acid binding protein S1; RNA binding protein S1, serine-rich domain	RNPS1_HUMAN	1-23	1-161	SRP54	14729963
SEC31 homolog A (S. cerevisiae)	SC31A_HUMAN	876-989	800-1113	PDCD6	17196169
SH2B adaptor protein 1	SH2B1_HUMAN	1-313	1-555	JAK2	16824542

SIL1 homolog, endoplasmic reticulum chaperone (S. cerevisiae)	SIL1_HUMAN	152-215	1-256	HSPA5	18840615, 16282978
Suppressor of cytokine signaling 7	SOCS7_HUMAN	285-319	124-494	SORBS3	15242778
**Intramolecular Interactions**					
Charged multivesicular body protein 3	CHMP3_HUMAN	16-35	2-113	CHMP3, C-terminus	19525971
**Signals**					
Mitochondrial antiviral signaling protein	MAVS_HUMAN	40-97	10-77	NLRX1 motif	19692591
Nuclear receptor coactivator 6	NCOA6_HUMAN	972-1964	1491-1495	LXRapha, ERalpha	17908797
SH2B adaptor protein 1	SH2B1_HUMAN	1-313	146-152	Nuclear localizatio n signal	21486950

Table S3B: Genes with tissue-specific exons that map to protein interfaces. Residues in Tissue-specific Cassette exons that were involved in forming an interface in a protein complex are listed. The interfaces that were formed between the protein with TS segment and other proteins, DNA or RNA in the complex were examined. The column 'Protein ID' shows UniProt protein identifiers for the proteins with TS segments, the column 'PDB ID' gives PDB identifiers of the examined complexes, the column 'Chain' shows names of the reference chains in the PDB structures. Finally positions of residues in PDB structures, which form both predicted biologically relevant interface and crystal-contact interface (as obtained from the PISA database) are given. All residues listed in the table are obtained from the PISA database, and those amino acid residues for which difference in accessible surface area (ASA) between complex and monomer in a PDB biological unit was greater than 10% of the amino acid radius, i.e. residues that are more likely to have a role in biological interactions, are marked with a star. (ASA was calculated with Areaimol (http://www.ccp4.ac.uk/html/areaimol.html) and criteria for interface definition was taken from (Levy, 2010)).

Protein	PDB structure with TS segment	Reference chain with TS segment	Residues in the corresponding chain that are encoded by TS segments and that are present in the interface (crystal and/or subunit interface,* in the structure)
CDKN3	1FPZ	A	11,18,41
RPB4	2C35	A	12,13,16,17,18,19,24,27,28,30*,31*,45,47,50,51,54,57,58*, 60*
PPM1K	2IQ1	A	3,4,8,23,24,26,50,54
IL6RA	1N26	A	253,254,255,264,265,274,279,283,284,292*,294*,296*, 297*
THIK	2IIK	A	103*,106*,107*,109
PUM1	1IB2	A	177,178,181,188,189
MKNK1	2HW6	A	8,11,12,13,14,15,16,19,21,24,31,32
RLA2	2W1O	A	2*,3,6,9*,10,13*,14,27,30*,31,33
GSTT1	2C3N	A	129,133,136,140,149,160,161
AK1A1	2ALR	A	198,200,201,202,217,219,223,226,230
CO2	3ERB	A	5,17,22,24,41,44,45,47,51
DPOLB	1BPX	A	26,30,31,34*
UBE2K	1YLA	A	96,101
GAK	3LL6	A	148
GSTT1	2C3N	A	50,61,63,64*,65*
MAVS	2VGQ	A	412,415,416,438,439,442,445,448,454,457,458,461
CO2	3ERB	A	57,59,64,74,75,76,79,93
ODPA	1NI4	A	144*,145,148*,149,151*,152,155*,156,158,172*,173
PPT1	3GRO	A	219,222,224,225
SAE2	1Y8Q	B	49,50,52,53,56,59,60
BGLR	1BHG	A	169,172
DHPR	1HDR	A	34,35,38,45,47,48,50,52,53
ALKB2	3H8O	A	42,43,46,49,50,51,52,53,54,57,59,72,73,92
FES	3BKB	A	1,4,61,62
BGLR	1BHG	A	443,447,450
THTPA	3BHD	B	11,19,21,22,23,27,28,29,30,34,38,54,56,57,67,70,71,82, 84,85,95,97,98,99,100,102,105,114,115,129,130,131, 135,136,137,138, 139,148,150,151,161,162,163,164, 165,167,168,171,175,178
GMFG	3L50	A	35,41,43,44,45,46
NMNA3	1NUP	B	123,156,165,173
SYWC	2AZX	A	312
SUMF2	1Y4J	A	86,87,88,89
THIK	2IIK	A	60,61,62,63,66,67,70,71,74,75
ARL1	1UPT	A	3,4,29
NQO2	1ZX1	B	103,104*,105*,106*,109,112,116,128,130,131*
RAB4B	2O52	A	55,56,58,60,63
GGA1	1OXZ	A	111,115
GLYM	3OU5	A	41,42,44,45,47,48,49,50,51,52,53,54,55,222

CSAD	2JIS	A	278,281,288
TYPH	2J0F	A	2,4,7,8,11,13,15,32,34,35*,36,37*
ABHEB	1IMJ	A	1,14,15,29,40,41,49,50,54,55,58
CSAD	2JIS	A	112,113,114,116*,117,120*,124,127,131,134*,136,137, 144
MBNL1	3D2Q	D	1,16,33,58
MACD1	2X47	A	80,82,86
FPPS	1YQ7	A	237,243,248
MGN	2HYI	A	49,51*,53*,54,57,60,61,81
FABD	2C2N	A	155,158,159,160,161,163
SRR	3L6B	A	8,12,15,16,27,47
DHPR	1HDR	A	140,144,148,151*,152,155*,159*,160,161,162*
BGLR	1BHG	A	173,174,178
CCND3	3G33	B	47,50,52,53,90,91,96,99,113*,116
UBE2K	1YLA	A	152*,156*,169,173,174,178
UPP1	3EUE	A	59,62,63,64,65,66,67,68,69,71,72,73,79
MASP1	3DEM	A	2,3,5,12,36,38,39,46,47,49,50
HMDH	1DQ8	A	80,81,82,83,85,87,88,89,90,91,92,94,106,112,113,114, 117,121
PHF8	3KV4	A	59,104,145,177,184,424,430
SAE1	1Y8Q	A	251,253,254
ERCC1	2A1J	B	20,21,24,31,34,35,38,39,41
CLM8	2Q87	A	2,4,5,7,9,13,15,17,22,23*,36,37,38,49,56,74,76,77,78,89,91, 92,94,95*,96*,107
SAT2	2BEI	A	65,66,67,68,70,71,72,74,79*,85*,86*
AOFB	1GOS	A	46,90
GSK3B	1I09	A	329,334,337,338
RED1	1ZY7	A	12,35,36,38,113,125,184,250,371,374
UCK2	1XRJ	A	149,159,163,167*,171
MGLL	3JW8	A	157,161,162,164,165,166,169,170,171,172,173,183,184
ASAP1	2D1X	C	1,2,9,60
THYN1	3EOP	A	127,133,146,148,156
DOK1	2V76	B	2,4,8,9,10,11,14,15,17,19,21,25,38,39,40,41,42,43,44,45,52, 56,60,61
UBE2K	1YLA	A	57*,59,66,67,70,71
VINC	1TR2	A	618,624,688
RUNX1	1E50	C	4,6,7,9,18,27,104,105
CRYL1	3F3S	A	92,107,108,110,111
RFFL	1Y02	A	23,24,25,28,29,50,54,55,58,66,68,69,71,73,76,77,78,83, 86,87,88,89,91
DHR11	1XG5	C	211,215,224
TRMB	3CKK	A	42,51,53,57,60,61,63,64,65,67,69,93
BIN1	2FIC	A	37,51,53
ZDH17	3EU9	A	2,3,4,6,8,9
PTPA	2HV6	A	1,2,4,7,10,12,13,16,19,20
U2AF1	1JMT	A	8,14,20
ARLY	1AOS	A	1,2,5
SDSL	2RKB	A	74,77,79,90,91,94,95,97,98,100,102,104,105*,106
TBRG1	2WZO	A	1,2,3,4,5,7,8,9,15,19
FKBP5	1KT0	A	171,176,177,180,181,186,187
DUS10	2OUC	A	6,10,13,23,35,36,38,40,41,42,43,44,45,47,53,57,58,61,62, 63,64,65, 68,69,72,82,88,90,91,92,93,104,105,106
MKNK1	2HW6	A	203,204,208,223,224,226
SAR1A	2GAO	B	33,35,36,37,39
SIRT6	3PKI	A	11,12,14,31,45,46
THIK	2IIK	A	242,258,261,263,264,271,273,275,295
FLT3L	1ETE	A	110,113,114,118,121,122,123,126,131,133,134
NQO1	1D4A	A	104*,105*,106*,110*,113*,117*,128*,132*,134,138
TRMB	3CKK	A	4,6,15,16,21,29

CHLE	2PM8	A	66,68,71,72,84,99,102*,155,293,297,298,299,302*,306, 308*,361,368,374,375,450,454,455,473*
IMDH2	1B3O	A	89,93,96
I13R1	3BPN	C	48,49,75,77*,83,93
DUS22	1WRM	A	49,51,52
PDLI1	2PKT	A	35,36,37,46,47,55,56,57,64,68,70,73,76,77
GAK	3LL6	B	136,176,177,178,179,180,185,186,187
LAP2	1N7T	A	37
PACN2	3HAJ	A	165,166,169,252,256
GALE	1EK5	A	291
PTGR2	2ZB4	A	191,192,195,196,199,210,211,219,223,244
IF4A2	3BOR	A	52,69,70,71,72,84,87
PEX19	3AJB	B	11,12,14,15,18,20,22,24*,25*,26*
TRI37	3LRQ	A	9*,10,13*,14,17,18,19,21,24,25,27*,28,30*,32*,33,38,42
CLK2	3NR9	B	2,3,4,5,6,7,8,12,13,17,19,23,26
ATOX1	1FE0	A	10,11*,12*,13,16,17,20,21*
PUR8	2J91	A	396,397,398,399,400,401,408,412,434,435,438,439
UBA3	3GZN	I	31,35
EFNA1	3CZU	B	115,116,117,118
EPHB4	2VWV	A	8,113,120,121,124,164
PP1A	3EGG	A	15,17,18,20*,23*,25,26,27,35,42
DECR	1W6U	A	1,2,5,9,10,13,17
RGN	3G4E	A	126,127,128,138,139,140,144,169,171*,180,181,184
PI51C	3H1Z	P	4*,7,8*,9*,10*,11*,12*,13*,14*,15*
PF21A	2PUY	A	5,6,9,10,11,12,13,14,15,16,18,20,29,32,33,34,36,37,38, 40,41,44,49
VRK3	2JII	A	57,61,65,68,69,72,74
GALE	1EK5	A	1,2,24,25,36,40
CLK2	3NR9	B	2,3,4,5,6,7,8,12,13,17,19,23,26
ILK	3F6Q	A	31,32,34,42,47,50,51,54,55,64,66*,76,79
CDK2	1AQ1	A	142,157,158,160
NAGK	2CH5	A	17,34,36,41
PM14	2F9D	A	1,2,8,9,13*,14,17,18,21,22,25,26,29,33,35,36*,37,38
BCAT2	1EKF	A	11,13,14,16,17,30*,31*,32*,34*,42,50,52,56*,58*,59*, 60*,61*,62*,63*,68*,69*,70*,71*,73*
MBNL1	3D2N	A	7,10,12,14,16,18,20,21,22,26,30,31,32,39,45,48
CHMP3	3FRT	A	21
EXOS8	2NN6	C	1,10,11
ETFA	1T9G	R	11,12,27
COAC	1QZU	A	15,18
PTPA	2HV6	A	10,93,94,246,261
SETMR	3BO5	A	35,37,42,46,49,52,54,55,59,60,61,62,67,74,81,82,84,91, 92,94,100,111,118,119,127,128,146,147,150,151,154, 155,180,219,220,221,231,235,237,242,248,249,250,254, 256,258,259,261
BIRC6	3CEG	A	189,190,196,198,199,202
KAPCA	3AGL	A	247,250,268,269,276,286,300
ZFYV9	1DEV	B	4,6,7,9,10,12,13,14,15,16,17,18,19,20,21,27,28,32,33,34,35, 36,37,38,39,41
PYRD	1D3G	A	168,169
NOVA1	2ANN	A	38,42,52
ACK1	1U46	B	87,88,89,90,98,130,131,147,149,150,152,155,161
GOSR2	3EG9	A	60,62,140,143
KKCC2	2ZV2	A	255
BGLR	1BHG	A	292
GLO2	1QH3	A	122,123,125,126,127

Table S3C: List of tissue-specific exons that encode PTM sites. Representative isoforms with TS Cassette exons that had a high difference in the TS exon inclusion levels (switch score on the scale of 0 to 1 was greater than 0.5) between two different tissue types (cell lines are excluded from this table) and also encoded PTM sites are listed. Positions of TS protein regions in the protein sequences encoded by the 'Representative transcripts' are shown in the column 'TS segment'. Gene name, tissues with the highest difference in inclusion levels and original scores for the exon inclusion in each tissue (that range from 0 to 1), as well as the original switch-score (which summarizes the difference in inclusion levels) are also shown. Finally, positions of annotated PTM sites in TS segments are given. Since control calculations in the original analysis were performed on six different cerebellum samples, the highest number of differential exon inclusion events was reported for this tissue.

Representative transcript	Gene	Tissue 1	Tissue 2	Switch-score	TS segment	Amino acid positions of annotated PTM sites
ENST00000341928	*EPB41L3*	cerebellum	adipose	0.91	784-824	787,784,788
ENST00000263246	*PACSIN2*	breast	cerebellum	0.88	343-383	373,375,370,372
ENST00000335312	*PIP5K1C*	cerebellum	lymph node	0.88	641-668	649
ENST00000264447	*ZNF638*	cerebellum	liver	0.81	1-439	128,420,383
ENST00000345122	*ARHGAP5*	cerebellum	lymph node	0.80	1-1239	968,1173,1124,1138,1195,765,1115,1202,1218,1176,1129,550
ENST00000400013	*TJP1*	brain	testes	0.80	922-1001	927,968
ENST00000373447	*KIAA0406*	breast	liver	0.72	1-767	459
ENST00000337526	*RTN4*	cerebellum	breast	0.70	205-1004	860,446,863,361,881,889
ENST00000341928	*EPB41L3*	colon	adipose	0.69	1052-1087	1081
ENST00000312239	*HP1BP3*	lymph node	adipose	0.67	1-32	6
ENST00000397501	*PTK2B*	cerebellum	lymph node	0.66	739-780	762,758,765
ENST00000291552	*U2AF1*	heart	lymph node	0.64	45-66	59
ENST00000298406	*NAA30*	cerebellum	brain	0.63	1-257	152,190,39,199,196,117,55
ENST00000355634	*SORBS2*	brain	heart	0.62	308-834	371,750,376
ENST00000374580	*BMPR2*	breast	cerebellum	0.59	529-955	757,586,863,680,681
ENST00000366899	*DUSP10*	cerebellum	lymph node	0.58	1-270	4
ENST00000374796	*NCOA6*	cerebellum	liver	0.56	972-1964	1321
ENST00000303648	*C12ORF32*	cerebellum	brain	0.54	1-56	33
ENST00000389862	*ADARB1*	cerebellum	brain	0.54	10-321	26
ENST00000340281	*ZNF326*	cerebellum	brain	0.53	70-205	137
ENST00000379446	*NEDD9*	breast	liver	0.52	5-153	92,12
ENST00000278520	*CCDC82*	cerebellum	adipose	0.50	1-262	154,198,192,195
ENST00000302979	*POLR1D*	breast	brain	0.50	1-8	1

Table S4A,B,C, related to main figure 5

Table S4A: List of tissue-specific exons that encode predicted interaction motifs. Representative isoforms with TS Cassette exons that encode protein interaction motifs and have a high difference in the TS exon inclusion levels (switch score [as described in Wang et al, on the scale of 0 to 1] was greater than 0.5) between two different tissue types are listed. Cell lines are excluded from the list. Positions of TS protein segments in the protein sequences encoded by the 'Representative transcripts' are shown in the column 'TS segment'. Gene name, tissues with the highest difference in inclusion levels and original scores for the exon inclusion in each tissue (that range from 0 to 1), as well as the original switch-score (which summarizes the difference in inclusion levels) are also shown. Finally, positions of predicted binding motifs in the TS segments are given. Since control calculations in the original analysis were performed on six different cerebellum samples, the highest number of differential exon inclusion events was reported for this tissue.

Representative transcript	Gene	Tissue 1	Tissue 2	Switch-score	TS segment	Predicted binding motifs
ENST00000356443	*MYOM1*	skel. muscle	testes	1	836-931	888-904,867-879,839-847
ENST00000312827	*MLF1*	heart	testes	1	82-96	83-94
ENST00000330274	*MAP7D2*	brain	testes	1	72-104	81-104
ENST00000259238	*BIN1*	adipose	skel. muscle	1	255-269	255-269
ENST00000348159	*MEF2D*	adipose	skel. muscle	0.98	286-292	286-292
ENST00000345434	*FHL1*	cerebellum	skel. muscle	0.98	230-296	278-288
ENST00000331495	*FMNL1*	cerebellum	lymph node	0.98	1071-1100	1087-1094,1071-1077
ENST00000300843	*MARK4*	cerebellum	testes	0.97	626-652	637-652,626-630
ENST00000378292	*TPM2*	colon	skel. muscle	0.97	188-213	201-210
ENST00000406818	*DTNB*	cerebellum	testes	0.96	519-525	519-522,525-525
ENST00000358025	*SYNE2*	skel. muscle	testes	0.95	6445-6467	6445-6467
ENST00000361166	*NF2*	skel. muscle	testes	0.95	580-590	584-590
ENST00000293590	*FMNL3*	colon	lymph node	0.95	318-347	333-340,318-324
ENST00000395072	*SYNGAP1*	brain	lymph node	0.93	765-778	765-778
ENST00000357980	*TPM1*	colon	heart	0.93	39-80	70-78
ENST00000325888	*FLNC*	adipose	skel. muscle	0.93	1734-1766	1734-1736
ENST00000341360	*ATP2B4*	cerebellum	adipose	0.92	1104-1162	1135-1142
ENST00000341928	*EPB41L3*	cerebellum	adipose	0.91	784-824	792-819
ENST00000380503	*INO80E*	breast	skel. muscle	0.91	157-161	157-161
ENST00000310418	*CLTB*	cerebellum	breast	0.9	155-172	155-172
ENST00000330909	*CNKSR3*	breast	brain	0.89	1240-1269	1255-1264
ENST00000335312	*PIP5K1C*	cerebellum	lymph node	0.88	641-668	641-661
ENST00000263246	*PACSIN2*	cerebellum	breast	0.88	343-383	383-383,343-366
ENST00000369046	*QRSL1*	lymph node	testes	0.87	186-244	186-187
ENST00000344290	*MAPT*	skel. muscle	testes	0.86	45-73	45-56,58-73

ENST00000171887	*TNS1*	breast	heart	0.86	1000-1007	1000-1001
ENST00000354329	*MYO18A*	cerebellum	skel. muscle	0.84	1951-1965	1951-1958
ENST00000361941	*SORBS1*	heart	skel. muscle	0.83	602-635	607-618
ENST00000315939	*LOC100132369*	cerebellum	adipose	0.83	945-1037	1034-1037,997-1010,967-974
ENST00000344237	*EPB41L1*	brain	testes	0.82	58-69	67-69
ENST00000262965	*TCF3*	breast	colon	0.82	529-607	564-580,587-602
ENST00000333577	*EP400*	adipose	testes	0.81	515-550	520-550
ENST00000264447	*ZNF638*	cerebellum	liver	0.81	1-439	170-180,204-229,108-121,137-152,1-19,383-394,256-271,29-94
ENST00000389759	*PKP4*	cerebellum	testes	0.81	1043-1085	1048-1057,1070-1079
ENST00000345122	*ARHGAP5*	cerebellum	lymph node	0.8	1-1239	993-1024,1201-1212,1046-1067, 1091-1127,931-938,965-978, 1153-1170
ENST00000400013	*TJP1*	brain	testes	0.8	922-1001	922-958,965-1001
ENST00000309868	*ABLIM3*	cerebellum	adipose	0.8	402-434	428-434,411-417
ENST00000374389	*SRRM1*	cerebellum	lymph node	0.79	538-551	538-551
ENST00000355815	*SREBF1*	brain	lymph node	0.79	31-60	51-60,31-33
ENST00000297164	*RELL2*	brain	lymph node	0.79	84-105	103-105
ENST00000376454	*KIAA1217*	adipose	skel. muscle	0.78	560-594	569-589
ENST00000289968	*ARHGAP17*	cerebellum	skel. muscle	0.78	497-574	548-572,497-539
ENST00000323460	*SENP5*	cerebellum	brain	0.77	1-504	306-317,330-344,385-394,183-200,451-476,107-119,366-376,256-267,224-251,133-144,410-418
ENST00000242285	*CLTA*	cerebellum	breast	0.77	180-191	180-187
ENST00000397983	*MAP2K7*	skel. muscle	testes	0.76	42-57	42-57
ENST00000361941	*SORBS1*	skel. muscle	testes	0.75	552-579	566-575
ENST00000268676	*DEF8*	cerebellum	lymph node	0.75	1-57	32-45
ENST00000276420	*DOK2*	breast	lymph node	0.74	22-115	106-114
ENST00000355394	*ABI1*	cerebellum	lymph node	0.73	361-389	373-389,361-364
ENST00000317968	*PDLIM5*	breast	skel. muscle	0.73	98-236	166-221,107-136
ENST00000376142	*ABI1*	cerebellum	lymph node	0.72	155-159	155-159
ENST00000394023	*SMARCC2*	brain	colon	0.71	563-593	575-588,593-593

ENST00000361941	*SORBS1*	cerebellum	breast	0.71	148-270	183-215,227-236,257-270,153-171
ENST00000337526	*RTN4*	cerebellum	breast	0.7	205-1004	516-525,763-783,385-393,839-847,420-429,205-209,457-484,583-600,570-581,541-563,663-672,799-807,713-723,283-292,220-258,406-412,744-753,611-621,686-707
ENST00000315939	*LOC100132369*	skel. muscle	testes	0.69	792-944	819-827,792-792
ENST00000344749	*TCF3*	breast	adipose	0.69	529-604	582-599,532-538,565-578
ENST00000336686	*LRRFIP2*	skel. muscle	testes	0.69	489-522	495-500
ENST00000316724	*BIN1*	cerebellum	skel. muscle	0.69	422-457	456-457,422-452
ENST00000335141	*FNBP1*	cerebellum	lymph node	0.69	331-391	367-391,331-337
ENST00000397661	*NFIX*	brain	skel. muscle	0.69	319-359	348-356,323-338
ENST00000341928	*EPB41L3*	adipose	colon	0.69	1052-1087	1067-1077,1052-1059
ENST00000346183	*NFATC3*	cerebellum	breast	0.67	35-412	74-80,39-49,225-235,309-335,277-288,180-207,349-358,148-154,388-396,110-116
ENST00000374222	*UBXN11*	cerebellum	testes	0.67	34-66	38-45,50-60
ENST00000375590	*C1ORF144*	cerebellum	lymph node	0.67	18-33	19-30
ENST00000312239	*HP1BP3*	adipose	lymph node	0.67	1-32	1-32
ENST00000368128	*EPB41L2*	breast	brain	0.66	870-910	870-874
ENST00000397501	*PTK2B*	cerebellum	lymph node	0.66	739-780	739-751,768-779
ENST00000246912	*MLX*	adipose	testes	0.65	81-110	81-100
ENST00000306729	*ASPSCR1*	lymph node	testes	0.65	452-545	535-545,487-500,462-469
ENST00000367303	*RMND1*	cerebellum	testes	0.65	1-168	114-127
ENST00000339995	*EIF4G2*	cerebellum	liver	0.64	434-471	434-471
ENST00000317268	*SEP-04*	brain	lymph node	0.63	21-119	69-78,21-28,89-99,30-48
ENST00000357634	*SAMD4A*	cerebellum	skel. muscle	0.63	238-325	238-255,264-291
ENST00000298406	*NAA30*	cerebellum	brain	0.63	1-257	22-41,59-73,1-12,83-116,125-170, 184-197
ENST00000355634	*SORBS2*	brain	heart	0.62	308-834	512-518,334-339,693-706,468-477,775-783,755-765,448-456,793-

						832,723-741,353-359,545-556,312-324,610-617,573-583,653-672
ENST00000229395	*FGFR1OP2*	breast	skel. muscle	0.62	133-170	143-152
ENST00000355842	*ADD1*	cerebellum	lymph node	0.61	652-662	652-662
ENST00000344715	*NR1H3*	cerebellum	breast	0.61	15-77	52-67,32-44,15-26
ENST00000396916	*HCFC1R1*	heart	liver	0.61	32-50	32-37
ENST00000298687	*NDRG2*	cerebellum	skel. muscle	0.61	26-39	29-34
ENST00000371138	*STX16*	breast	lymph node	0.61	133-186	161-170,133-140
ENST00000319080	*MLXIP*	cerebellum	breast	0.6	334-364	334-350
ENST00000395932	*PALM*	adipose	brain	0.6	234-277	234-243,253-277
ENST00000336498	*ARHGAP10*	lymph node	skel. muscle	0.59	676-726	704-716,676-690
ENST00000374580	*BMPR2*	cerebellum	breast	0.59	529-955	572-609,685-694,770-861,540-570,729-745,622-664,868-893,702-717,939-955,909-925
ENST00000340692	*MOCS1*	breast	lymph node	0.59	368-383	382-383
ENST00000298694	*FLJ10357*	breast	lymph node	0.59	1415-1462	1439-1445,1453-1462
ENST00000401644	*SNED1*	adipose	lymph node	0.59	1273-1305	1287-1297,1273-1280
ENST00000395736	*TOM1L2*	brain	colon	0.58	431-450	433-450
ENST00000366899	*DUSP10*	cerebellum	lymph node	0.58	1-270	25-31,50-55,228-233,144-154
ENST00000395547	*ATXN2L*	brain	heart	0.58	1047-1097	1060-1074,1047-1053
ENST00000406818	*DTNB*	breast	brain	0.57	579-608	579-608
ENST00000396637	*PEX5*	brain	testes	0.57	215-251	215-221
ENST00000377245	*TJP2*	breast	colon	0.57	998-1107	1074-1107,1003-1025,1033-1054
ENST00000378004	*ARHGAP26*	cerebellum	breast	0.56	663-699	675-682
ENST00000355341	*ZFYVE19*	cerebellum	breast	0.56	276-343	276-283,326-337,305-315
ENST00000374796	*NCOA6*	cerebellum	liver	0.56	972-1964	1900-1930,1818-1841,1214-1223, 1028-1208,1236-1326,1472-1537, 1597-1602,1883-1895,1776-1801, 1857-1875,1940-1964,1549-1565, 972-998,1655-1668,1349-1427, 1694-1709,1620-1628,1727-1769, 1436-1455

ENST00000375005	*TBRG1*	colon	lymph node	0.56	152-197	186-194
ENST00000376454	*KIAA1217*	adipose	skel. muscle	0.56	1179-1204	1200-1204,1179-1189
ENST00000355634	*SORBS2*	brain	colon	0.56	112-126	120-126
ENST00000377245	*TJP2*	cerebellum	breast	0.55	961-997	971-986
ENST00000373921	*C1ORF38*	breast	testes	0.55	216-573	421-429,571-573,368-375,389-397,531-546
ENST00000309315	*ZMIZ2*	cerebellum	lymph node	0.55	332-357	332-357
ENST00000395813	*CAST*	heart	skel. muscle	0.54	91-112	91-112
ENST00000173898	*TRO*	cerebellum	brain	0.54	16-412	40-58,142-147,262-268,288-297,367-399,166-185,319-339,101-118,214-224,16-18,195-200
ENST00000389862	*ADARB1*	cerebellum	brain	0.54	10-321	38-47,59-72,137-145,76-116,10-29
ENST00000323926	*FN1*	cerebellum	brain	0.54	2083-2171	2136-2149,2087-2129
ENST00000303648	*C12ORF32*	cerebellum	brain	0.54	1-56	11-39,50-56
ENST00000379999	*FBXO18*	adipose	testes	0.53	52-103	71-76
ENST00000401952	*DLGAP4*	brain	testes	0.53	671-696	671-696
ENST00000331320	*MTA1*	cerebellum	testes	0.53	593-604	594-604
ENST00000340281	*ZNF326*	cerebellum	brain	0.53	70-205	154-161,83-95,107-122,184-194
ENST00000338834	/	brain	colon	0.53	138-168	138-144
ENST00000379446	*NEDD9*	breast	liver	0.52	5-153	43-48
ENST00000288398	*TPM1*	breast	colon	0.52	258-284	261-268
ENST00000245441	*NIN*	cerebellum	lymph node	0.52	800-1512	1205-1214,934-939,1028-1038, 955-961,983-998,1225-1231, 1495-1502,1100-1116
ENST00000359188	*ABI1*	breast	brain	0.51	274-331	274-331
ENST00000369353	*LOC652164*	breast	lymph node	0.51	2192-2271	2267-2271
ENST00000072644	*YIPF1*	liver	testes	0.51	11-65	11-29
ENST00000273146	*FAM198A*	cerebellum	liver	0.51	1-356	1-21,161-173,90-136,47-67
ENST00000348077	*TOX2*	adipose	lymph node	0.5	330-461	354-396,440-454,407-431
ENST00000389044	*TRIP12*	adipose	brain	0.5	33-74	33-49,51-74
ENST00000355394	*ABI1*	breast	brain	0.5	302-360	302-324,328-360
ENST00000375799	*PLEKHM2*	brain	skel. muscle	0.5	218-237	218-231

Table S4B: List of kinases that contain a tissue-specific segment with predicted interaction motifs but no detectable Pfam protein domains. Kinase genes, which, depending on the tissue of expression differentially include protein segments with interaction motifs embedded in disordered regions, are listed. TS segments that overlap protein domains are not shown. Columns in the table show representative transcripts for each of the TS exons, kinase gene names, protein coordinates of the TS segments, switch-scores that indicate difference in exon inclusion levels between the two tissues (scale 0 to 1), pairs of two tissues with the highest differential exon inclusion levels and positions of predicted interaction motifs in the TS segment. Since control calculations in the original analysis were performed on six different cerebellum samples, the highest number of differential exon inclusion events was reported for this tissue. The list of kinases was obtained from www.kinome.org.

Representative transcript	Kinase gene name	TS segment	Switch score	Tissues with differential exon inclusion levels	Position of predicted binding motif
ENST00000300843	*MARK4*	626-652	0.97	cerebellum and testes	626-630, 637-652
ENST00000324219	*MAP4K4*	623-699, 569-622	0.88, 0.93	brain and MB435, HME and brain	569-581, 583-622, 685-692, 623-675, 696-699
ENST00000381916	*TNK2*	578-592	0.85	skel. muscle and T47D	587-592
ENST00000315939	*LOC100132369*	792-944, 945-1037, 714-741	0.69, 0.83, 0.25	skel. muscle and testes, cerebellum and adipose, T47D and HME	714-715, 724-735, 819-827, 792-792, 967-974, 1034-1037, 997-1010
ENST00000397983	*MAP2K7*	42-57	0.76	skel. muscle and testes	42-57
ENST00000355999	*STK39*	415-435	0.66	MCF7 and MB435	415-430
ENST00000397501	*PTK2B*	739-780	0.66	cerebellum and lymph node	739-751, 768-779
ENST00000374580	*BMPR2*	529-955	0.59	breast and cerebellum	572-609, 685-694, 770-861, 729-745, 540-570, 622-664, 868-893, 702-717, 939-955, 909-925
ENST00000402010	*MARK2*	472-525	0.58	T47D and lymph node	472-525
ENST00000392334	*CSNK1D*	400-409	0.46	MCF7 and lymph node	400-409
ENST00000297954	*WNK2*	2076-2108, 679-730	0.46, 0.25	heart and colon, testes and cerebellum	679-711, 2099-2108
ENST00000361168	*CLK2*	134-161	0.44	colon and BT474	150-155
ENST00000344096	*DYRK2*	17-66	0.43	colon and breast	47-66, 17-41
ENST00000297532	*FASTK*	28-168	0.43	cerebellum and colon	33-66
ENST00000368361	*CLK2*	134-162	0.38	adipose and BT474	151-156
ENST00000375300	*SIK3*	366-413	0.38	testes and brain	375-383
ENST00000324771	*DDR1*	505-541	0.37	MCF7 and adipose	505-521, 539-541
ENST00000322680	*CAMK2G*	385-422	0.36	brain and T47D	385-391, 417-422
ENST00000355280	*MINK1*	594-630	0.35	breast and testes	594-611
ENST00000333602	*TNK2*	965-994	0.33	HME and cerebellum	977-986, 992-994, 965-969
ENST00000351936	*FGFR2*	56-144	0.31	T47D and cerebellum	136-144
ENST00000395749	*CAMK2B*	316-340	0.3	cerebellum and	334-340

				skel. muscle	
ENST00000316626	*GSK3B*	379-411	0.28	MCF7 and cerebellum	384-401, 379-379
ENST00000392473	*CAMKK2*	72-248	0.27	MCF7 and MB435	152-160, 130-150, 245-248, 200-220
ENST00000318588	*CASK*	603-614	0.27	adipose and lymph node	614-614

Table S4C: List of transcription factors that contain a tissue specific segment with predicted disordered regions but no detectable Pfam protein domains or predicted binding motifs. Transcription factor (TF) genes, which - depending on the tissue of expression - differentially include exons that encode disordered regions, are listed. TS segments that overlap protein domains are not shown. The columns show representative transcripts for each of the TS exons, TF gene name, protein coordinates of the TS segment, switch-scores that indicate difference in exon inclusion levels between the two tissues (scale 0 to 1), names of the two tissues with differential exon inclusion levels and positions of predicted disordered regions in the TS segment. Since control calculations in the original analysis were performed on six different cerebellum samples, the highest number of differential exon inclusion events was reported for this tissue. List of TFs were obtained from published literature (Vaquerizas et al., 2009).

Representative transcript	TF Gene	TS segment	Switch-score	Tissues with differential exon inclusion levels	Disordered regions in TS protein segments
ENST00000348159	*MEF2D*	286-292	0.98	skel. muscle and adipose	286-288
ENST00000317578	*SIX5*	268-536	0.78	BT474 and skel. muscle	268-296, 361-418, 422-425, 428-428, 459-462, 465-468
ENST00000407404	*NR1H3*	1-14	0.71	adipose and T47D	1-9
ENST00000359486	*TCF20*	1934-1960	0.69	cerebellum and lymph node	1940-1960
ENST00000346183	*NFATC3*	35-412	0.67	breast and cerebellum	35-38, 45, 96-98, 101-103, 109-110, 119-147, 152-152, 162-164, 203-209, 211-277, 282-311, 355-355, 361-363, 365-382, 399-399, 389-389, 401, 406, 410-412
ENST00000404876	*TCF20*	235-250	0.66	cerebellum and lymph node	239-250
ENST00000228251	*CSDA*	192-260	0.66	MB435 and skel. muscle	192-260
ENST00000246912	*MLX*	81-110	0.65	testes and adipose	100-110
ENST00000344715	*NR1H3*	15-77	0.61	breast and cerebellum	15-77
ENST00000319080	*MLXIP*	334-364	0.6	breast and cerebellum	353-364
ENST00000330387	*CREB3L2*	35-106	0.57	breast and MB435	63-88, 90, 94, 96-104
ENST00000340281	*ZNF326*	70-205	0.53	cerebellum and brain	70-73, 77-78, 91-93, 100-101, 104-107, 120-122, 126-126,130-131, 133-135, 143-145, 147-150, 156-160, 165-205
ENST00000373953	*ZNF76*	444-498	0.51	cerebellum and MB435	447-448, 457-458, 465-466, 468-476, 482-482 489-491
ENST00000374685	*RXRB*	79-161	0.49	cerebellum and MCF7	79-161
ENST00000379540	*NFX1*	9-344	0.44	cerebellum and skel. muscle	9-10, 22, 26-38, 41-63, 65-168, 170-174, 177-178, 189-300, 336-337, 313-315, 318, 321-322, 324-330
ENST00000322733	*ARNT*	140-154	0.44	MB435 and MCF7	140-154

ENST00000330243	*IRF7*	165-239	0.42	colon and testes	165-190, 192-192, 217-239
ENST00000264110	*ATF2*	35-66	0.42	cerebellum and testes	64-66
ENST00000283629	*UBP1*	274-309	0.41	brain and lymph node	281-300, 303-303, 307-309
ENST00000340699	*PBX1*	333-370	0.4	brain and testes	333-338
ENST00000235372	*PRDM2*	171-207	0.39	breast and lymph node	171-207
ENST00000298282	*PKNOX2*	1-29	0.38	brain and cerebellum	1-29
ENST00000392348	*LOC731605*	800-848	0.38	cerebellum and BT474	800-805, 810-840, 846-846
ENST00000216923	*ZFP64*	96-149	0.35	MCF7 and MB435	109-110, 112-114, 118-118, 121-138
ENST00000372922	*TRERF1*	546-628	0.35	HME and MCF7	546-581, 595-628
ENST00000362042	*NFE2L1*	261-290	0.34	colon and cerebellum	261-265
ENST00000348066	*CAMTA2*	1-10	0.34	cerebellum and skel. muscle	1-9
ENST00000394679	*ZNF207*	187-202	0.32	BT474 and testes	187-189, 191-195, 199-200
ENST00000398899	*ERG*	232-255	0.32	lymph node and adipose	247-255
ENST00000396801	*ZNF384*	102-117	0.31	colon and lymph node	102, 104-117
ENST00000333640	*POU6F1*	1-15	0.31	colon and cerebellum	1-7
ENST00000222305	*USF2*	77-143	0.3	skel. muscle and heart	118-121, 125-135
ENST00000374012	*PHF20*	428-520	0.3	breast and testes	482-520
ENST00000272369	*MEIS1*	397-415	0.3	cerebellum and testes	397-398, 402, 410-415
ENST00000277225	*ZNF462*	2079-2142	0.28	testes and MB435	2121-2142
ENST00000355467	*ZNF512*	11-29	0.27	cerebellum and HME	11-29
ENST00000219069	*ZNF263*	130-189	0.26	MCF7 and cerebellum	130-131, 134-189
ENST00000334521	*ZNF410*	377-423	0.25	colon and testes	377-387, 392-402, 404-411, 423
ENST00000375200	*ZNF341*	48-113	0.25	lymph node and testes	100-107
ENST00000350777	*HINFP*	1-60	0.25	BT474 and colon	1-7, 43-60

Table S5A,B, related to main table 1

Table S5A: Genes associated with embryonic lethality and genes that were found to be mutated in cancer are enriched in the genes with tissue-specific exons. Genes with tissue-specific isoforms were compared to all other human genes mapped to mouse orthologs that had one or more phenotypes associated to it (as obtained from the Mouse Genome Informatics - MGI database) or to all other human genes with Hugo nomenclature (HGNC) identifiers (as obtained from the Ensembl version 54), in order to asses whether the genes associated with embryonic lethality or genes found to be mutated in cancer, respectively, were enriched in the genes with tissue-specific isoforms. The column N_{total} shows the number of genes that were successfully mapped to the identifiers in the underlying disease gene databases. The column N_{+} shows the number of genes with tissue-specific isoforms or all other genes in the database that were implicated in the disease, as applicable, and N_{-} those that were not annotated as such. Genes that were found mutated in cancer cells were obtained from the COSMIC database. p-values were calculated with the Chi-Square test in R.

Analysis	Set of exons	N_{+}	N_{-}	N_{total}	p-value
**Genes associated with embryonic lethality**	With tissue-specific isoforms	202	963	1,165	$p<1.2x10^{-8}$
	Other human genes	2,080	15,722	17,802	
**Census Cancer Genes**	With tissue-specific isoforms	31	1,153	1,184	$p<6.2x10^{-2}$
	Other human genes	345	18,630	18,975	
**Genes that were found mutated in cancer cells**	With tissue-specific isoforms	227	957	1,184	$p<3.2x10^{-6}$
	Other human genes	2,697	16,278	18,975	

Table S5B: List of domains that are most frequently present in proteins with tissue-specific segments but that do not overlap tissue-specific segments. Domains that are most frequently encoded by transcripts with tissue-specific exons, but not by tissue-specific exons themselves, are listed. Percentage of proteins with at least one copy of a particular domain is denoted for the proteins with tissue-specific segments and is compared to proteins that contain segments encoded by Other Cassette or Constitutive exons. When the difference in frequencies of proteins that contain the concerned domain is significant (Chi-square test) p-value is shown.

Domain	Domain name	Frequency in TSE genes (not overlapping with TS segment)	Frequency in all other genes	p-value
**RRM_1**	RNA recognition motif	2% (27/1,310)	0.9% (157/21,828)	$p<5.8x10^{-6}$
**SH3_1**	SRC homology 3 domain	2% (24/1,310)	0.7% (113/21,828)	$p<10^{-7}$
**Pkinase**	Protein kinase domain	2% (21/1,310)	2% (314/21,828)	/

SUPPLEMENTAL EXPERIMENTAL PROCEDURES

Exon dataset

TS Cassette exons were composed by mapping cassette exons with differential tissue inclusion levels, as reported by Wang and co-workers, to Ensembl protein coding exons (release 54; www.ensembl.org). Wang *et al.* reported exons as differentially included if the exon inclusion and exclusion levels, calculated from sequencing reads that mapped to the exon, surrounding exons and exon junction, significantly differed between at least two tissues. The tissues investigated include adipose, brain, breast, cerebellum, colon, heart, liver, lymph node, skeletal muscle and testes; as well BT474, HME, MB435, MCF7 and T47D cell lines. In our study, a TS exon was mapped to a previously annotated exon if the difference between the coordinates (i.e. start or end) of the reported TS exons and coordinates of known and predicted Ensembl protein coding exons was at most two nucleotides. The set of Other Cassette exons was composed from the protein coding Ensembl exons that fulfilled the following criteria: the exon was (i) alternatively present in at least two transcripts with its whole length, (ii) not mutually exclusive with an adjacent exon and (iii) there was another RNA isoform that also contained different exons upstream and downstream of the exon of interest. The latter was a necessary condition to avoid cases of alternative start and termination sites. Exons that overlapped with TS Cassette exons were excluded from the set of Other Cassette exons. It should be noted that the set of Other Cassette exons used here might contain as yet uncharacterized tissue-specific exons. A set of Constitutive exons was composed from the protein coding Ensembl exons that were present in all transcript products with their whole length and with unchanged boundaries. Each exon was associated with the longest protein-coding transcript that contained it. Only transcripts with two or more exons were considered in the study.

Mapping of Pfam domains

We used Pfam software (Finn et al., 2010) to predict protein domains in all representative protein sequences. We then analyzed how often protein segments encoded by three different exon datasets overlapped a protein domain, or encoded a full protein domain (approximated with at least 90% of domain length, since domain borders are frequently not precise). We found that the protein segments encoded by both TS Cassette and Other Cassette exons overlapped a protein domain less frequently than Constitutive exons: 44% (or 628/1426) of TS Cassette exons encoded at least a small portion of domain sequence (mostly less than 10% of domain sequence); similarly 44% (or 6216/13,755) of the segments encoded by Other Cassette exons overlapped a domain, while 55% (or 75355/137,046) of Constitutive exons overlapped domain sequence. This suggests Constitutive exons more often encode protein domains than TS Cassette and Other Cassette exons ($p<2.2x10^{-16}$ for both comparisons, Chi-square test). However, when a protein domain already overlapped a TS segment, it was more common that the segment contained a whole domain compared to when a domain overlapped a segment encoded by Other Cassette or Constitutive exons: 11% (or 72/628) of domain overlaps with a TS segment were cases of a segment containing the full domain, compared to 8% (or 489/6216) for Other Cassette and 8% (or 6088/75355) for Constitutive exons; $p<10^{-3}$ and $p<10^{-3}$, Chi-square test.

Disorder prediction with the VSL2 method

To control for the potential bias in the disorder prediction method, we also carried out prediction of disordered regions using the VSL2B software (Peng et al., 2006). VSL2B is a baseline predictor of the VSL2 method, which uses support vector machine method for the prediction of disordered residues. The method is trained on well-characterized disordered proteins and the VSLB2 application takes into account only amino acid composition of a protein. This makes it faster than the main VSL2 method and hence it is recommended for genome-scale studies (Peng et al., 2006). Since the prediction method recognizes only symbols for standard 20 amino acids, we removed all non-standard symbols (positions with ambiguously assigned amino acids) from the sequences. After the prediction was carried out, we assigned to the removed amino acids, the same status as the surrounding amino acids (disorder or order) had. Prediction of intrinsically disordered residues by this method confirmed that protein segments encoded by Tissue-specific exons were significantly enriched in disordered residues compared to the segments encoded by Other Cassette and Constitutive exons. The fraction of TS exons that encoded intrinsically disordered proteins segments was 53% (756/1426), which was significantly higher than the fraction of Other Cassette – 43% (5958/13,755) or Constitutive – 35%(47533/137,046) exons that encoded disordered segments; $p<2.6x10^{-12}$ and $p<2.2x10^{-16}$, respectively, Chi-square test.

Control calculations for exon length and dataset size

Datasets of exons that we composed for the comparison with Tissue-specific exons were of different size than the set of TS Cassette exons, and exons in them did not have the same distribution of lengths as Tissue-specific exons. To control whether our observations were independent of these dataset traits, we composed smaller

subsets of Other Cassette and Constitutive exons that were closer to the traits of Tissue-specific exons. Distribution of lengths of Tissue-specific exons did not follow the normal distribution ($p<2.2x10^{-16}$, Shapiro test), and the median length of the encoded protein segments was 44 amino acids, which was longer than the median lengths of the segments encoded by Other Cassette and Constitutive exons: 34 and 41 amino acids, respectively. We therefore composed sets of Other Cassette and Constitutive exons that had the same median length of the encoded protein segments as Tissue-specific exons: for this we excluded all exons in the complete sets that encoded segments which were shorter than 25 and 21 amino acids, respectively. This left us with 9,074 Other Cassette and 120,412 Constitutive exons. From each of these datasets, we randomly chose 1,426 exons, i.e. we composed exon datasets of the same size as the set of Tissue-specific exons and noted the characteristics of the protein segments encoded by these exons: fraction of disordered segments, fraction of segments with an interaction motif and fraction of segments with a PTM site (**Tables S1E**). We repeated the random sampling procedure 1,000 times. To compare the distribution of values for the random subsets of filtered Other Cassette and Constitutive exons with the fraction of TS segments that were disordered, had a binding motif or a PTM site, we used cumulative probability distribution function in R (Ross Ihaka, 1996). P values for each of the examined characteristics were calculated as $P (X >= x) = f (x, \mu, \sigma)$ where $f (x, \mu, \sigma)$ was the probability density function of the normal distribution with mean μ and standard deviation σ. μ and σ were estimated from the random subsets of exons. The density function thus provides the probability that a value greater than or equal to x is observed by chance, given the observed distribution of values. Normal distribution of values in the randomly chosen subsets of exons was examined with the Shapiro-Wilk test in R. Results of this analysis showed that TS segments were indeed enriched in all of the examined characteristics. Results, together with the associated p-values, are in **Table S1E**.

Calculation of conservation of exons in the three different sets of exons

For the representative protein sequences with segments encoded by TS Cassette, Other Cassette and Constitutive exons, we compared conservation between human and mouse protein segments as well between DNA regions encoding these segments, i.e. coding residues in these exons. For the comparison, only one-to-one human-mouse orthologs were considered and this information was obtained from Ensembl 54, which was initially used for mapping exon coordinates to protein segments. Coding DNA and protein sequences of protein pairs were aligned with Needleman-Wunsch algorithm (from the Emboss package: http://emboss.sourceforge.net/, default settings both for protein and DNA alignments). Conservation values were analyzed only for segments longer than 10 amino acids which were present in the orthologous mouse proteins; criteria for this was that more than 90% of the segment was aligned to the orthologous mouse sequence. For these segments, we calculated percentage of identical residues in mouse orthologs from protein pairwise alignments. We also performed the same calculation for predicted disordered regions in these protein segments only, as well as a separate analysis for the predicted binding motifs and all other residues in these protein segments. We performed analogous calculations for the corresponding residues in DNA alignments. Distribution of values between different groups was compared with Mann-Whitney test in R.

Next, we extracted segments from the protein alignments that corresponded to the analyzed exons, and we also extracted DNA sequence regions that encoded the aligned human and mouse protein segments. We performed reverse alignment from the R seqinr package to align DNA sequences based on the protein alignments and used kaks function in the R seqinr package to calculate Ka and Ks values for each segment. Additionally, for tissue-specific segments that contained predicted binding motifs, we divided residues based on whether they belonged to a motif or not and calculated Ka and Ks values for these sub-regions in TS segments.

Measurements of network centrality in TSE-containing genes

The use of a network formalism to describe biological interactions enables the use of many tools from graph theory. In particular, it permits us to assess the "topological importance" that a single node (e.g. protein) has in an interaction network. For instance, what will be the impact of removing a given protein from the network? How critical is a protein to maintain a given pattern of interactions? Such notion is encapsulated by the concept of "centrality". Several approaches can be used to assess the centrality of a node in a network. In this case, we used four independent metrics to test whether protein-coding genes with Tissue Specific cassette Exons (TSE genes) tend to occupy central position in the human protein-protein interaction networks (PPI networks). All the following metrics were computed for the entire human protein-protein interaction network, using the "iGraph" package for R (http://igraph.sourceforge.net/).

Betweenness centrality (btc) measures the extent to which a given node connects other nodes in the network, thereby forming a path (Gursoy et al., 2008). Considering a node *v* in a network, btc gives the proportion of

shortest paths from all nodes to all others that pass through v. The betweenness centrality of a node v is therefore defined as:

$$btc(v) = \sum_{s \neq v \neq t} \frac{\sigma_{st(v)}}{\sigma_{st}}$$

where σ_{st} gives the total number of shortest paths between a node s and a node t (both distinct from v), and $\sigma_{st}(v)$ is the number of such paths that include v.

Closeness centrality (C_C) of a node v is the inverse of "farness", that is the total distance of this node to all other nodes in graph. The further apart a node is from the other nodes in the graph, the closer to zero is its closeness centrality (Sabidussi, 1966). More formally, closeness centrality is defined as:

$$C_c(v) = \sum_{t \in V} 2^{-d(v,t)}$$

where $t \in V$ denotes any node belonging to the network, and $d(v,t)$ the shortest distance between v and t.

PageRank. The PageRank algorithm is commonly known as a core component of the Google search engine (Sergey Brin, 1998), but its usefulness and relevance for biological networks has been recently illustrated (Ivan and Grolmusz, 2011), and implementations of the algorithm can be found in statistical network analysis packages such as iGraph. In the context of a protein-protein interaction network, the algorithm iteratively assigns a score to each protein based on the connectivity of neighboring nodes it is connected with. A protein will have a high Page rank if it is interacting with many highly connected proteins.

Kleinberg's Hub score. This algorithm, based on the concept of 'hubs and authorities' as defined by Kleinberg (Kleinberg, 1999; Kleinberg, 2000) is a precursor of the PageRank algorithm. Given a graph G, the hubs of G are determined through the adjacency matrix A of G, by computing the eigenvectors of the product AA^T. Based on this, the relative importance of each protein is computed taking into account the hub score of its interacting partners. The more hubs are connected to a protein, the higher is its score.

These four metrics were computed for all individual proteins of the human protein-protein interaction network, and the distributions of (i) TSE genes with predicted binding motif, (ii) other TSE genes and (iii) genes without TSE were compared, using a non-parametric test (Mann-Whitney test). Results of each measurement are provided in **Figure S3A**.

Jaccard similarity index and the comparison of tissue-specific interaction landscapes of TSE and non-TSE genes

Genes with tissue-specific splicing isoforms were taken from Wang et al, 2008. By definition, such genes have one isoform containing a cassette exon that is significantly more included (in relative abundance, compared to the other isoforms of the same gene) in some tissues than in others. These tissue-specific splice isoforms are therefore considered either "included" or "excluded" in a given set of tissues. Bossi and Lehner, integrated robust multi-array averaged expression profiles of human genes with protein-protein interactions data to generate a series of tissue-specific protein-protein interaction networks. In this study, the authors assembled a high confidence dataset of known protein-protein interactions based on low throughput and high throughput experiments. The authors discretized tissue-specific mRNA expression levels and accordingly removed non-expressed genes from their protein-protein interaction network of reference, thereby generating tissue-specific interactomes. Among the 10,230 genes present in the protein interaction network, 8,235 had information on tissue-specific interactions. In particular, more than 60% of TSE genes (n=740) had information on tissue-specific interactions.

Dataset construction

To perform the interaction similarity analysis, we restricted the analysis to tissues overlapping between both Wang et al, 2008 and Bossi and Lehner, 2009 studies, which resulted in 15 human tissues. Noticeably, the correspondence of tissue types across the two studies is incomplete. In particular, the tissue-specific interactomes depict a more detailed repertoire of tissues than the measurements of differential expression of tissue-specific exons. Hence, we restricted our analysis to tissues that could unambiguously be related in both studies, resulting in 9 tissues from Wang et al, 2008 (out of 15) and we related these to 15 tissues from Bossi

and Lehner, 2009 (out of 79). In particular, it was assumed that the inclusion levels of tissue-specific exons that were quantified in whole brain and whole testes Wang et al, 2008 – were valid for substructures within testes (i.e. seminiferous tubules, Leydig cells, germ and interstitial tissues) and brain (temporal lobe, cingulate cortex, prefrontal cortex, thalamus).

Principle of the calculation
We used the Jaccard similarity index to quantify the extent to which the interaction partners of a gene are maintained in a pair of tissue. The Jaccard similarity coefficient for a gene *g* expressed in a pair of tissues *A* and *B* was defined as:

$$J_{A,B}(g) = \frac{|\cap_{A,B} PPI(g)|}{|\cup_{A,B} PPI(g)|}$$

where $\cap_{A,B}PPI(g)$ denotes the common interaction partners of *g* in tissues *A* and *B*, and $\cup_{A,B}PPI(g)$ denotes the all possible interactions partners of *g* in tissues *A* or *B*. Values span between 0 and 1, where 0 indicates that the interaction partners of *g* in *A* and *B* are strictly different, and a value of 1 indicates that interactions partners of *g* in *A* and *B* are strictly identical.

Data processing
We applied the calculation for all genes in the human protein-protein interaction network, in all pairwise combinations of tissues included in the analysis (**Figure S3B**). We then selected Jaccard similarity indices of genes and pairs of tissues for which in each compared tissue at least one interaction was found, thereby removing ambiguous cases were a gene appears to have tissue-specific partners (Js=0) simply because it is either not expressed in the two tissues, or because none of its interaction partners are expressed.

We then selected the Jaccard similarity indices of TSE genes in the pairs of tissues where isoform inclusion levels were found to be significantly different ("TSE genes", **Figure 3D**). We also selected the Jaccard similarity scores of non-TSE genes in the same pairs of tissues used for the "TSE genes" set ("non-TSE genes", **Figure 3D**). Further, we selected the Jaccard similarity indices from "TSE genes" for genes with predicted binding motifs of at least 10 amino acids ("TSE with motif", **Figure 3D**), and those genes without such motif ("TSE without motif", **Figure 3D**).

Monte-Carlo simulation
To test if the observed distributions of Jaccard similarity indices could be expected by chance, we performed a Monte-Carlo simulation employing 100,000 trials, where we randomly re-assigned Jaccard similarity indices to the genes, and computed how many times the random expected mean value of Jaccard indices of a given set of genes was lower than the real mean value observed in the original set. We apply such calculation for 3 sets of genes: (1) non-TSE genes, (2) TSE genes, and (3) TSE genes that contain a predicted binding motif of at least 10 amino acids (**Figure S3C**).

Information entropy of interaction patterns across different tissues
Since TSE genes showed a higher average number of interaction partners in the consensus human protein interaction network (**Figure 3A**), as well as in tissues sub-networks where tissue-specific alternative splicing has been observed (**Fig 3B**), we investigated whether the interaction partners of TSE genes tend to vary across tissues using another independent metric. We took advantage of existing tools from information theory to quantify how variable are the interaction partners of a gene across the 79 human tissues Bossi and Lehner integrated in their study (Bossi and Lehner, 2009).

Principle of the calculation
A simple formalism was used to quantify heterogeneity in the interaction partners that protein-coding genes encounter across multiple tissues, and is illustrated in **Figure S3D** (left panel).

The status of a given interaction across many tissues can conveniently be described by a vector, made of 1 and 0s that denote the presence or absence of that interaction; each position in the vector corresponding to a defined tissue. Hence, looking at the *N* interactions partners of a given gene across *M* tissues simply requires considering *N* vectors of size *M*, made of 1 and 0s.

More formally, considering the interactions of a gene: $(ppi_1, \ldots, ppi_N)$ the N vectors are defined as:

$$\begin{cases} \boldsymbol{v_1} = \left(\mathbf{1}_{tissue\ 1}(\boldsymbol{ppi_1}), \mathbf{1}_{tissue\ 2}(\boldsymbol{ppi_1}), \ldots, \mathbf{1}_{tissue\ M}(\boldsymbol{ppi_1})\right) \in \{\mathbf{0},\mathbf{1}\}^{\boldsymbol{M}} \\ \vdots \\ \boldsymbol{v_N} = \left(\mathbf{1}_{tissue\ 1}(\boldsymbol{ppi_N}), \mathbf{1}_{tissue\ 2}(\boldsymbol{ppi_N}), \ldots, \mathbf{1}_{tissue\ M}(\boldsymbol{ppi_N})\right) \in \{\mathbf{0},\mathbf{1}\}^{\boldsymbol{M}} \end{cases}$$

where $1_{tissue}(ppi)$ equals 1 if the interaction is present in the tissue, and 0 if not.

In this context, measuring variations of the N interaction partners across multiple tissues becomes equivalent to measuring the dissimilarity of the N vectors all at once. Importantly, one must be able to distinguish not only the number of interactions a gene has in a given tissue (from 0 to N), but also the exact identity of these interactions. An efficient way to maintain such information is to take, for each tissue, the status of each interaction (0 or 1), and form a "letter" with it.

$$\boldsymbol{l_{tissue\ 1}} = \left(\boldsymbol{v_1}(\boldsymbol{tissue\ 1}), \ldots, \boldsymbol{v_N}(\boldsymbol{tissue 1})\right) \in \{\mathbf{0},\mathbf{1}\}^{\boldsymbol{N}}$$

For instance such a vector l_{tissue}^{gene} = "01010" can describe the status of 5 interaction partners of a gene in a given tissue. Hence, a gene will possess M letters in total, and the total number of unique letters for that gene will be $p \leq M$. If we note $(\theta_1, \theta_2, \ldots, \theta_p)$ the probability of apparition of each letter, then the Shannon entropy can be used to quantify how diverse the letters are, for each gene that has information for tissue-specific interactions. The Shannon entropy is given by:

$$\boldsymbol{H}(\boldsymbol{gene}) = -\sum_{i=1}^{p} \boldsymbol{\theta_i} \log(\boldsymbol{\theta_i})$$

The higher the Shannon entropy, the more letters one finds in the message, i.e. the more unique sets of interaction partners one finds across the analyzed tissues. We therefore referred to this metric as "interaction entropy". From this calculation we note that TSE genes with binding motifs have higher interaction entropy compared to TSE genes without binding motifs and non-TSE genes (**Figure S3D,** right)

Data processing

Genes that had one unique interaction partner across all tissues were removed from the analysis. Since the more interaction partners a gene has, the higher the number of unique words can be generated. Hence, a positive association between entropy and node degree is expected, and was indeed observed (r=0.678, $p<2.2\times10^{-16}$, Spearman rank correlation, **Figure S3D**, middle). We therefore scaled interaction entropy values on a running median to compare the interaction entropy of genes with different node degrees (**Figure S3D**, middle). As expected, this transformation drastically reduced the correlation between partnership entropy and node degree (-0.047, Spearman rank correlation). Distributions of interaction entropy (and scaled interaction entropy) for the analysed sets of genes were then compared with a Mann-Whitney test (**Figure S3D**, right) and the differences were found to be significant even after the scaling step. All calculations were performed in the R statistical package, and interaction entropy was computed with the help of the package "entropy" (Xing and Lee, 2006).

Analysis of functional enrichment

DAVID (http://david.abcc.ncifcrf.gov) was used to investigate functional enrichment in the different exon categories. Phenotype annotations for mouse genes and the list of genes associated with embryonic lethality were obtained from the Mouse Genome Informatics database (www.informatics.jax.org). The cancer gene census and genes from the COSMIC database (release 43) were downloaded from the corresponding databases at www.sanger.ac.uk/genetics/CGP/. Signaling pathway information was obtained from http://signalink.org.

Tissue-specific disordered regions in transcription factors and kinases

A list of human kinases was obtained from www.kinome.org. Of the 506 investigated human kinases, 45 had a TS segment. Kinases for which TS segments did not overlap a Pfam domain but contained interaction motifs embedded in disordered regions (25 kinases) are provided in Table S14B. A set of human transcription factors (1,391 TFs) was obtained from the literature (Vaquerizas et al., 2009) and those TFs that contained TS segments were further investigated (67 TFs). Pfam protein domains were mapped to the encoded protein sequences and those TS segments that did not overlap with any Pfam domains or predicted binding motifs but contained disordered regions are listed in **Table S4C** (40 TFs).

SUPPLEMENTAL REFERENCES

Finn, R.D., Mistry, J., Tate, J., Coggill, P., Heger, A., Pollington, J.E., Gavin, O.L., Gunasekaran, P., Ceric, G., Forslund, K., *et al.* (2010). The Pfam protein families database. Nucleic Acids Res *38*, D211-222.

Gursoy, A., Keskin, O., and Nussinov, R. (2008). Topological properties of protein interaction networks from a structural perspective. Biochem Soc Trans *36*, 1398-1403.

Ivan, G., and Grolmusz, V. (2011). When the Web meets the cell: using personalized PageRank for analyzing protein interaction networks. Bioinformatics *27*, 405-407.

Kleinberg, J.M. (1999). Authoritative sources in a hyperlinked environment. J. ACM *46*, 604-632.

Kleinberg, J.M. (2000). Navigation in a small world. Nature *406*, 845.

Levy, E.D. (2010). A simple definition of structural regions in proteins and its use in analyzing interface evolution. J Mol Biol *403*, 660-670.

Peng, K., Radivojac, P., Vucetic, S., Dunker, A.K., and Obradovic, Z. (2006). Length-dependent prediction of protein intrinsic disorder. BMC bioinformatics *7*, 208.

Ross Ihaka, R.G. (1996). R: a language for data analysis and graphics. Journal of Computational and Graphical Statistics *5*, 299-314.

Sabidussi, G. (1966). The centrality of a graph. Psychometrika *31*, 581-603.

Sergey Brin, L.P. (1998). The Anatomy of a Large-Scale Hypertextual Web Search Engine. Proceedings of the 7th World-Wide Web Conference, Brisbane, Australia.

Sickmeier, M., Hamilton, J.A., LeGall, T., Vacic, V., Cortese, M.S., Tantos, A., Szabo, B., Tompa, P., Chen, J., Uversky, V.N., *et al.* (2007). DisProt: the Database of Disordered Proteins. Nucleic Acids Res *35*, D786-793.

Vaquerizas, J.M., Kummerfeld, S.K., Teichmann, S.A., and Luscombe, N.M. (2009). A census of human transcription factors: function, expression and evolution. Nature reviews. Genetics *10*, 252-263.

Xing, Y., and Lee, C. (2006). Alternative splicing and RNA selection pressure--evolutionary consequences for eukaryotic genomes. Nature reviews. Genetics *7*, 499-509.
